# Genetic Analysis of Root-to-Shoot Signaling and Rootstock-Mediated Tolerance to Water Deficit in Tomato

**DOI:** 10.3390/genes12010010

**Published:** 2020-12-23

**Authors:** Maria J. Asins, Alfonso Albacete, Cristina Martínez-Andújar, Eser Celiktopuz, İlknur Solmaz, Nebahat Sarı, Francisco Pérez-Alfocea, Ian C. Dodd, Emilio A. Carbonell, Sevilay Topcu

**Affiliations:** 1 Instituto Valenciano de Investigaciones Agrarias, Carretera de Moncada a Náquera Km 4.5, Apartado Oficial, 46113 Moncada, Valencia, Spain; carbonell.emi@gmail.com; 2 CEBAS, CSIC, Campus de Espinardo, 30100 Espinardo, Murcia, Spain; alfonsoa.albacete@carm.es (A.A.); cmandujar@cebas.csic.es (C.M.-A.); alfocea@cebas.csic.es (F.P.-A.); 3 Faculty of Agriculture, University of Cukurova, 01330 Adana, Turkey; eceliktopuz@cu.edu.tr (E.C.); isolmaz@cu.edu.tr (İ.S.); nesari@cu.edu.tr (N.S.); stopcu@cu.edu.tr (S.T.); 4 The Lancaster Environment Centre, Lancaster University, Lancaster LA1 4YW, UK; i.dodd@lancaster.ac.uk

**Keywords:** drought, QTL analysis, candidate genes, cytokinins, manganese, transcription factors, MAPKKK cascade, *S. pimpinellifolium*, rootstock breeding

## Abstract

Developing drought-tolerant crops is an important strategy to mitigate climate change impacts. Modulating root system function provides opportunities to improve crop yield under biotic and abiotic stresses. With this aim, a commercial hybrid tomato variety was grafted on a genotyped population of 123 recombinant inbred lines (RILs) derived from *Solanum pimpinellifolium*, and compared with self- and non-grafted controls, under contrasting watering treatments (100% vs. 70% of crop evapotranspiration). Drought tolerance was genetically analyzed for vegetative and flowering traits, and root xylem sap phytohormone and nutrient composition. Under water deficit, around 25% of RILs conferred larger total shoot dry weight than controls. Reproductive and vegetative traits under water deficit were highly and positively correlated to the shoot water content. This association was genetically supported by linkage of quantitative trait loci (QTL) controlling these traits within four genomic regions. From a total of 83 significant QTLs, most were irrigation-regime specific. The gene contents of 8 out of 12 genomic regions containing 46 QTLs were found significantly enriched at certain GO terms and some candidate genes from diverse gene families were identified. Thus, grafting commercial varieties onto selected rootstocks derived from *S. pimpinellifolium* provides a viable strategy to enhance drought tolerance in tomato.

## 1. Introduction

Agriculture aims to provide food and nutritional security for human life. However, it is highly dependent on water availability, since plants with limited water supply have a reduced capacity to transpire and draw water and nutrients to the root surface, which limits photosynthesis and final crop yield [1]. Over 35% of world land’s surface is considered arid or semi-arid, and the percentage of our planet affected by droughts has more than doubled in the last 40 years [2]. Due to climate change, some regions (mainly Mediterranean basin, Central China and West Africa) will be much affected by changes in precipitation regimes with more frequent drought periods [3]. The development of drought-tolerant cultivars is one of the most relevant FAO proposals to plan for drought.

From an evolutionary point of view, plants have evolved a number of anatomical, developmental, biochemical and physiological adaptations to limit desiccation of vegetative tissues [4]. The genes involved in these adaptations could be unveiled by exploring wild genetic resources to gain knowledge and develop molecular breeding tools. The closest wild relative to domestic tomato is *Solanum piminellifolium* [5,6], which originated in Ecuador and expanded to northern and Southern Peru, where its niche space became more associated with cold and drought [7]. Therefore, it might be a better donor of drought tolerance than the well-known drought-tolerant *S. pennelli* [8]. However, discovering the fraction of wild genetic diversity conferring drought tolerance requires the phenotyping of large numbers of lines and long-lasting breeding programs to introgress genes into modern tomato varieties. In contrast, isolating and ectopic expression of genes can modulate plant drought tolerance by regulating transcription factors, hormonal balance, or plant metabolism [3,9,10,11,12,13]. Critical genes involved in abiotic stress tolerance have been identified and, in general, can be classified into two types: functional genes, encoding relevant enzymes and metabolic proteins, and regulatory genes, which would correspond to transcription factors, protein kinases, and protein phosphatases [10]. Various transcription factors are involved in the regulation of the ABA-dependent signaling pathway and play a major role in the stress response by regulating the expression of many downstream drought-responsive genes [3,14]. Other hormones, particularly cytokinin, affect the drought stress response [3,15]. Several transcription factors in different species, including crops, have been used to improve plant response to drought stress [3,10,13,16,17]. Mitogen-activated protein kinase (MAPK) cascades have been identified in various signaling pathways involved in plant development and stress responses [18]. Overexpression of *DSM1* (a *Raf-like MAPKKK* gene) in rice increased the tolerance to dehydration stress at the seedling stage [18]. The MAPK cascade also plays an important role in the drought stress response in horticultural plants, although the mechanisms by which it regulates plant stress resistance are largely unknown [19,20].

Since the global molecular, biochemical, and physiological plant response to drought will probably be different depending on the developmental stage and the intensity and duration of the water deficit [21,22], meta-analysis studies might be a highly valuable initial approach [23,24,25]. Thus, a meta-analysis of the responses of plants to water stress derived from 84 studies [25] revealed that this stress inhibits plant growth and photosynthesis and increases reactive oxygen species, plasma membrane permeability, and antioxidant activity. Noteworthy, plant roots were not significantly impacted by water stress in this study. It is also important to keep in mind that drought-tolerant, adapted accessions from different species may carry different sets of genes conferring such adaptation, making the genetic analysis of each species separately necessary. Thus, those generalized responses miss species-specific responses, and more importantly, for breeding purposes, the species-specific tolerance response.

To adapt to drought, plants regulate growth and development through long-distance chemical signaling (ABA, cytokinin, ethylene, peptides, increased xylem sap pH), allowing stomata closure to sustain shoot water status and the uptake of some ions against the nutritional stress [14,26,27,28]. Thus, xylem sap composition may be considered as a signal per se [21]. Besides, the strong relationship between elemental stoichiometry and metabolome reported by Rivas-Ubach et al. [29] could be explained by accumulating different metabolites depending on water availability [24,27]. Three physiological traits have been successfully targeted to improve drought tolerance in cereals and soybean: water-use efficiency (a measure of the ratio between the rates of photosynthesis and transpiration), stay-green (a heritable delayed foliar senescence), and reduction of stomatal density [1,30].

In tomato, several traits related to drought tolerance have been already studied through QTL (quantitative trait loci) analysis of natural genetic diversity from cultivated tomato [31,32] and the distant related wild species *S. pennellii* [33] and *S. habrochaites* [34,35]. *S. pimpinellifolium* have not yet been explored for this purpose.

Since roots regulate water uptake, grafting tomato varieties on improved rootstocks for water acquisition and translocation might increase water use efficiency without decreasing tomato yields and increasing nutrient fruit content [36,37,38]. Besides, grafting can delay leaf senescence, extending the harvesting period [39,40] in what could be considered as a stay-green trait. Grafting experiments help discern long-distance signaling [26] and to understand root function. Nevertheless, this approach has hardly been explored. Two large genomic regions in *S. pennellii* (IL8-3 and IL2-1) showed a rootstock-mediated effect on crop yield under drought [33]. The identification of rootstock-genomic regions (QTL) controlling drought tolerance related traits could allow marker-assisted selection in rootstock breeding programs and the search for new alleles in wild germplasm because, following Price [41], those QTLs are expected to contain the genes involved. Taking advantage of the complete tomato genome sequence by the Tomato Genome Consortium [42], and the availability of a large panel of SNPs (SolCAP panel, http://solgenomics.net/), genome assembly allows the rapid identification of candidate genes within 2 Mbp around the physical position of the SNP(s), with observed maximum LOD score (the QTL peak) and gain biological information from the QTL analysis.

Using a commercial variety grafted on a *S. pimpinellifollium* RIL population grown under well-watered and water-deficit conditions, this study aimed to (1) estimate the heritability of the rootstock effect on drought tolerance in terms of vegetative and flowering traits, and the phytohormone and nutrient xylem sap composition, (2) detect the QTLs involved and study their distribution and interactions, (3) disentangle the rootstock-dependent root-to-shoot communication and nutrient acquisition pathways, (4) investigate the genetic relationship of potential physiological components of rootstock-mediated drought tolerance, and (5) infer possible candidate genes for nutrient, hormone, and drought tolerance QTLs.

## 2. Materials and Methods

### 2.1. Plant Material, Growth Conditions and Trait Evaluation

This study used 123 F10 lines (P population) derived by single-seed descent from the hybrid between a salt-sensitive genotype of *Solanum lycopersicum* var. Cerasiforme (formerly *L. esculentum*) and a salt-tolerant line from *S. pimpinellifolium* L. (formerly *L. pimpinellifolium*) [43].

The commercial tomato hybrid *Solanum lycopersicum* cv. Boludo (named Bol) was used as scion, and plants from 123 lines of the P population were evaluated as rootstocks. Non-grafted (Bol) and self-grafted (Bol/Bol) plants were used as controls. Self-grafting placed a scion onto the roots of a different plant of the same genotype, and these controls were included to evaluate any physiological change caused by the grafting process per se.

Grafted plants having approximately six leaves were obtained from the seed company UNIGENIA Bioscience SLV (Murcia, Spain). Grafting was performed using the splicing method when seedlings had developed 3–4 true leaves. For that, seedlings were cut at the cotyledonary node, using the shoot as scion and the remainder as rootstock. Grafts were made immediately after cutting the plants, and grafting clips were used to adhere the graft union. Tomato plants were transplanted into a greenhouse of the Research Farm at the Faculty of Agriculture, Cukurova University (Adana, Turkey) on the 10–11 of October 2012, and all plants were irrigated just after transplanting. The greenhouse soil was sterilized and some physical and chemical properties of the soil were analyzed before starting the experiment. The same fertilizer amounts (NPK) were applied twice to all plants with irrigation before starting the water deficit treatment.

The greenhouse experiment was conducted in a split-plot design with three blocks; two watering treatments (well-watered, 100% of crop evapotranspiration, ETc, and water deficit 70% of ETc) as the main plots, and 123 graft combinations as sub-plots. Each plot consists of two plants of each graft combination, and the distances between rows and between plants within the rows were 80 and 50 cm, respectively. Plants were hung on wires running at 200 cm height over the rows. An automatic weather station located in the center of the greenhouse was used to estimate ET and irrigation water requirements. Irrigation was applied using a drip irrigation system and the irrigation interval was fixed at 7 days during the experiment.

On the transplanting and harvest days, soil water content was determined by using gravimetric soil samples. The access tubes of Aquacheck for weekly soil water content measurements were installed next to the plants for each graft combination in both irriga-tion treatments. Paired measurements of soil water content (Aquacheck, Cape Town, South Africa, Model AQMOB-X), stomatal conductance (Decagon Devices, Pullman WA, USA, SC-1 Leaf Porometer), and SPAD readings (Konica Minolta Sensing Inc., Osaka, Japan, SPAD 502 Plus) of each trial were taken occasionally.

Plants were harvested (blockwise) in the first week of December (after six weeks of treatment). Total shoot and leaf fresh (ShFW and LFW) and dry weights (ShDW and LDW) were determined (g) and used to estimate rootstock-mediated drought tolerance (ShFW_WD and LFW_WD). Two parameters of shoot water content were calculated: total shoot water content (ShWC in g) as the difference between ShFW and ShDW, and the proportion of water in the shoot (ShWp), as the proportion of ShWC to ShFW. The area of the fifth leaf in cm^2^ (LA) was also registered as well as the number of flowers (FlN) at the end of the experiment. One plant of each graft combination in each plot was cut above the graft union, and the spontaneously-exuding sap (under root pressure) was collected using silicon tubes and then stored in pre-weighted Eppendorf tubes. Sap flow rate was calculated using the exudation time (from 2 to 72 min) and the sap volume. Collected sap samples were immediately frozen in liquid nitrogen in the greenhouse and then stored at −80 °C. Xylem sap ionomic analysis determined Al, As, Be, Bi, B, Ca, Cd, Co, Cr, Cu, Fe, K, Li, Mg, Mn, Mo, Na, Ni, Pb, P, Sb, Se, S, Sr, Ti, Tl, V, and Zn concentrations (mg/L) using inductively coupled plasma—optical emission spectrometry (ICP-OES, ICAP 6000 Series, ThermoFisher Scientific, Waltham, MA, USA).

Cytokinins (trans-zeatin, tZ, trans-zeatin riboside, ZR, and isopentenyl adenine, iP), gibberellins (gibberellin A1, GA1, gibberellin A3, GA3, and gibberellin A4, GA4), indole-3-acetic acid (IAA), abscisic acid (ABA), salicylic acid (SA), jasmonic acid (JA), and the ethylene precursor 1-aminocyclopropane-1-carboxylic acid (ACC) were analyzed according to Albacete et al. [44] with some modifications. Briefly, xylem sap samples were filtered through 13 mm diameter Millex filters with 0.22 µm pore size nylon membrane (Millipore, Bedford, MA, USA). Ten µl of filtered extract were injected in a U-HPLC-MS system consisting of an Accela Series U-HPLC (ThermoFisher Scientific, Waltham, MA, USA) coupled to an Exactive mass spectrometer (ThermoFisher Scientific, Waltham, MA, USA) using a heated electrospray ionization (HESI) interface. Mass spectra were obtained using the Xcalibur software version 2.2 (ThermoFisher Scientific, Waltham, MA, USA). For quantification of the plant hormones, calibration curves were constructed for each analyzed component (1, 10, 50, and 100 µg L^−1^).

### 2.2. Statistical Analysis

A mixed model was used to assess the significance of each source of variation and to estimate the adjusted mean traits per rootstock genotype within each watering treatment for the QTL analysis, and to study the grafting effects by comparing Bol vs. Bol/Bol adjusted means.

Pearson correlation and principal component analyses were used to study associations between the different traits.

Broad sense heritability (H^2^) was calculated for traits measured in both populations assuming that the individuals from the F_9_ were nearly homozygous for all loci. Heritability was calculated as reported previously [45], using the formula: H^2^ = V_g_/(V_g_ + V_e_), where V_g_ and V_e_ are the estimates of genotype and environmental variance, respectively, by REML (Restricted Maximum Likelihood). These estimates were obtained by a model with the same sources of variation as above but considering rootstocks as random effects.

### 2.3. Molecular Markers and QTL Analysis

One hundred and thirty P-RILs at F_10_ were genotyped for 7720 SNPs from the SolCAP tomato panel (Illumina BeadXhip WG-401-1004) and a linkage map based on 1899 non-redundant SolCAP SNPs, covering 1326.37 cM of genetic length was used for QTL analysis [46].

QTL analyses of traits whose heritabilities were above 0.01 at least under one watering level were carried out using interval mapping (IM) and multiple QTL mapping (MQM) procedures in MapQTL ^®^ 6 [47]. A 5% experimentwise significance level was assessed by permutation tests. These LOD critical values ranged from 2.1 to 2.3, depending on the trait and chromosome. Significant QTLs were named by trait abbreviation (Table 1), the treatment where it was detected (Control, C, and Water Deficit, WD), the chromosome, and a number from 1 to 2 if more than one QTL was detected on the same chromosome for the trait and treatment concerned.

A two-way ANOVA was used to study the interaction (epistasis) between cofactors and markers corresponding to QTLs controlling the variation for the following traits: JA, B, and ShWC under control conditions, and B, ShWC, Mn, P, Mg, ABA, and ZR under water deficit.

Genes (ITAG2.4 gene models) covering 2.3–2.8 Mbp around the SNP(s) showing maximum LOD score at QTLs forming a cluster and QTLs governing the concentration of elements and phytohormones in the xylem sap were downloaded from the Sol Genomics Network (version SL2.50 at https://solgenomics.net/) and studied for function, root expression in the Heinz cultivar using the tomato eFP Browser (http://bar.utoronto.ca/efp_tomato/cgi-bin/efpWeb.cgi?dataSource=Rose_Lab_Atlas_Renormalized), and for the presence of frameshift InDels in the parental genomes using data reported by Kevei et al. [48]. Gene ontology (GO) enrichment analysis of genes within each QTL cluster region were carried out using the Singular Enrichment Analysis tool [49] at the AgriGo platform (http://systemsbiology.cau.edu.cn/agriGOv2/).

## 3. Results

**Figure 1:**
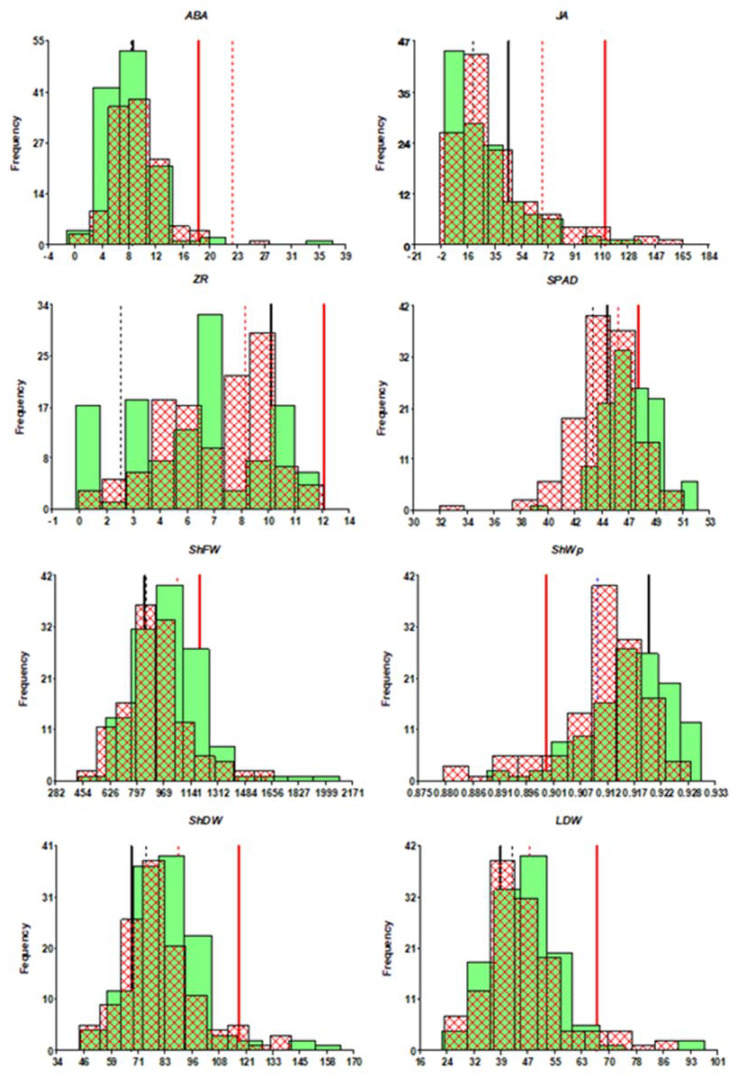
Distribution of relevant traits: ShDW, ShFW, LDW, SPAD, ShFW, ShWp, and xylem concentrations of ABA, JA, and ZR, under control (green-bar histogram) and water deficit (cross hatched-bar histogram). The position of controls Bol (black) and Bol/Bol (red) is indicated for control (thick bar) and water deficit (thin line). In the case of SHWp, the position of both controls under water deficit is the same (blue thin line).

To study rootstock effects on the tolerance of a tomato hybrid variety (cv. Boludo) to water deficit, we genetically analyzed several traits related to the vegetative/reproductive development, water content, and xylem concentration of phytohormones and nutrients. Comparing non-grafted and self-grafted plants (Bol and Bol/Bol, respectively) revealed that grafting per se increased ShFW, ShDW, LDW, SPAD, and xylem JA concentration, under well-watered conditions, while water deficit increased xylem ABA and ZR concentrations (Table S1 and Figure 1).

In general, grafting improved ShDW under control conditions, with over 84% of RILs as rootstocks, including the self-grafted controls, enhancing growth (Figure S1). Under water deficit, around 25% of RILs conferred larger ShDW than the self-grafted control. The same proportion of RILs conferred a larger degree of tolerance, measured as the proportional change in ShDW between watering levels (dShDW), than both controls. Interestingly, water deficit provoked a higher decrease of ShDW in self-grafted controls than in own-rooted plants. Besides, 30% of RILs conferred increased total shoot water content than controls when decreasing the irrigation level (dShWC).

The mean (and standard error) of phenotypic values observed for the analyzed traits in control lines, the range of variation in the RIL population, ANOVA results, and estimated broad-sense heritabilities under both irrigation regimes (C and WD), are presented in Table 1. Rootstock genotype (G) and the interaction rootstock genotype × irrigation regime (G × E) were significant for most traits. Notable exceptions were xylem sap K and As concentrations. LDW and ShDW were only significant for the rootstock genotype, while rootstock genotype and irrigation treatment affected ShWp. Heritability estimates of four drought tolerance traits (FlN, ShDW, ShFW, ShWC) increased notably under water deficit.

**Figure 2:**
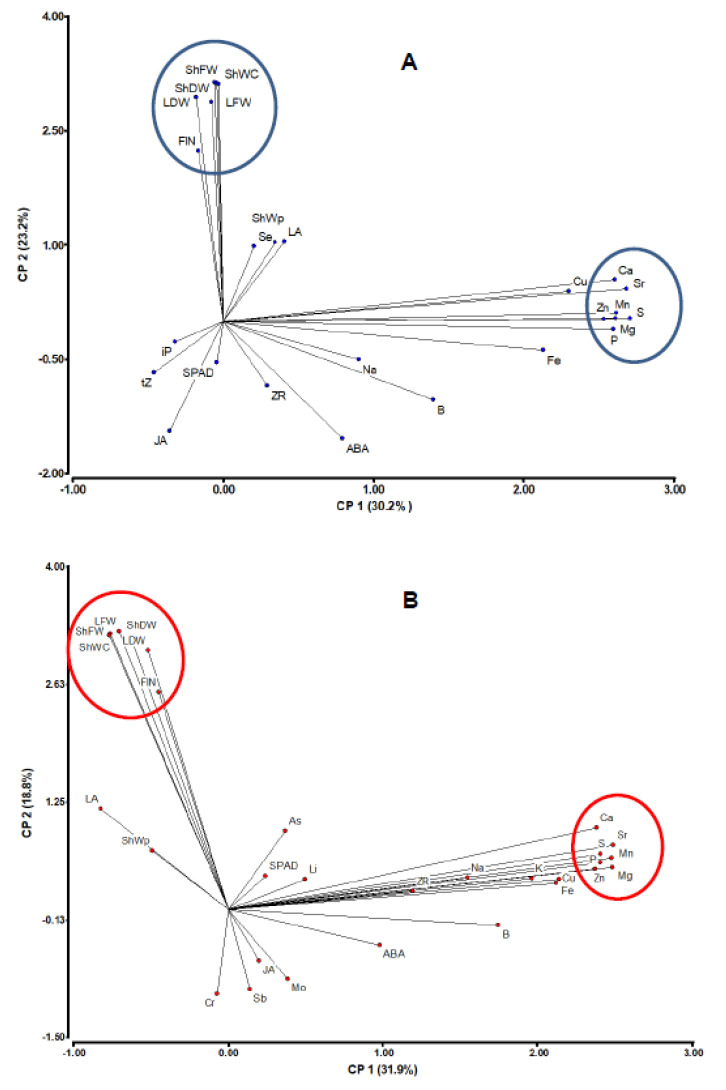
Graphic representations of principal component analysis of the traits correlation matrix based on the evaluation of RILs under control (**A**) and water deficit (**B**). The position of each heritable trait (see abbreviations in Table 1) is indicated by a point. Two groups of traits are noted (encircled).

Most vegetative traits (except for shoot water content, ShWC) were significantly correlated between irrigation regimes, while the xylem sap concentration of most analyzed components (except for ABA, Fe, and B) were not (Table S2). Genetic associations among traits were graphically represented by principal component analysis (Figure 2). Traits mostly contributing to the first component were xylem concentrations of Ca, Mg, Mn P, S, Sr, and Zn, while vegetative/reproductive traits (ShFW, ShDW, ShWC, LFW, LDW, and FlN) mostly contributed to the second component (Table S3). Reproductive and vegetative traits (related to drought tolerance under water deficit) were highly and positively correlated with shooting water content. Of the phytohormones, xylem ABA concentration was most highly (negatively) correlated with vegetative traits. Xyleme ABA concentration was most highly correlated with JA, particularly under control irrigation (*r* = 0.60; Table S2). The proportion of shoot water content to shoot fresh weight (ShWp) was significantly correlated with JA under control conditions, while LA and ZR were only correlated under water deficit. ShWC and ShWp were significantly correlated to only one hormone, the ABA, under water deficit, although correlation coefficients were relatively low (−0.22 and −0.21, respectively).

In total, 83 QTLs were detected, with most of them specific to one irrigation treatment (Table 2). The only exceptions were the “constitutive” QTLs Na_7 and FlN_4. In a few cases, QTLs for the same trait under both conditions were found to be linked, such as ShWC_4, ShWp_9, FlN_5, and FlN_8 (although with opposite gene effects). The genetic architecture of both traits related to the water content of the aerial part of the plant (ShWC and ShWp) were quite different. Only ShWp_C_3 and ShWC_WD_3 might be the same QTL although detected under different watering treatments. No QTL was detected for xylem concentrations of ACC, iP, As, Cr, Fe, K, Li, Mo, Sb, and Se. QTLs for ABA, ZR, Mn, P, S, Zn, Ca, and Cu were detected only under water deficit, while those for JA, tZ, and LA were exclusively detected under control conditions. Eleven significant epistatic interactions were detected for ShDW_WD (1), ShWC_WD (1), B_C (1), Mg_WD (2), JA_C (2), and Mn_WD (4), all of them similar to Mendelian-dominant epistasis in which one locus (cofactor or QTL marker) suppresses the allelic effects of a second locus (Figure S2).

There were 12 genomic regions (named in Latin numbers from I to XII) where QTLs of several traits are located together or form a cluster (Table 2 and Table S4). Thus, region V in chromosome 4, included QTLs for ShFW_C, LFW_C, ShDW_C, ShWC_C, LDW_C, FlN_C, FlN_WD, and ZR_WD; region X in chromosome 9, for ShFW_WD, ShWp_WD, FlN_WD, LFW_WD and ShWC_WD; and region XII on chromosome 5, for xylem ABA and ZR xylem concentrations under water deficit. Phytohormone and scion traits QTLs group together at region I (JA_C_1 and FlN_WD_1), V (ZR and vegetative and reproductive traits), and close to regions IX (ZR and drought tolerance traits) and XI (ZR and concentrations of Mn, Mg and P under water deficit).

The gene contents of 8 out of those 12 genomic regions were significantly enriched at certain GO terms (Figure S3): culling-RING ubiquitin ligase complex for cluster I; protein kinase activity and ubiquitin protein ligase binding for II; root development, plant cell wall, and pectin esterase inhibitor activity for III; cellular response to N starvation, negative regulation of transcription from RNA pol II promoter, and metal (non-S) cluster binding for IV; stomatal complex morphogenesis, cell wall pectin metabolic process, and extracellular space for V; negative regulation of stomatal opening, MAPK cascade involved in cell wall biogenesis, fungal type-cell wall organization, regulation of defense response by callose deposition, MAPKKK activity for VII; negative regulation of stomatal complex development, serine-type endopeptidase activity and apoplast for VIII; and cell recognition and rejection of self-pollen for X.

Using the criteria of molecular function (from gene annotation), the presence of frameshift InDels in mRNA coding sequence of parental genome [48], relative root expression (from Heinz), and ordinal gene number from gene 0 (the gene(s) containing SNP(s) with maximum LOD score) some putative candidate genes underlying QTLs governing xylem concentration of nutrients and hormones were prioritized (Table 3). Among them, several Cation/H^+^ antiporters, glutamate-gated kainate-type ion channel receptors, Mn and Mg transporters, Zn transporters, high-affinity sulfate transporter 1, and probable metal-nicotianamine transporter YSL7, were found for nutrient QTLs. Regarding phytohormones, several genes related to the biosynthesis (cytochrome P450, isopentenyl-diphosphate delta-isomerase, 9-cis-epoxycarotenoid dioxygenase 6, cysteine desulfurase, short-chain dehydrogenase/reductase and abcisic aldehyde oxidase) and metabolism (UDP-glycosyltransferase, zeatin O-β-D-xylosyltransferase, and cytokinin riboside 5′-monophosphate phosphoribohydrolase) of ABA and ZR were found within QTL regions governing the xylem concentration of these hormones.

In general, genes related to more than one signaling compound were found within QTLs for vegetative and flowering traits (clusters V, IX, III, and X) (Table S5). Thus, genes related to the auxin, ethylene, and gibberellin signaling occurred in the drought tolerance cluster IX; genes related to ABA, auxin, and cytokinin signaling were in the drought tolerance cluster III; and genes related to the ABA, ethylene, auxin, salicylic, and peptide signaling were in the drought tolerance QTL cluster X. A gibberellin receptor GID1L2 coding gene is in cluster V (Solyc04g079190), and the ABA transporter ABCG40 (Solyc09g091660), in cluster X. A gene coding for trehalose 6-phosphate phosphatase (Solyc03g083960) was found in cluster III, and the *phytaspase 2* (Solyc04g079360) at cluster V, which includes QTLs for the number of flowers.

Several transcription factors from DoF, WRKY, MYB, NAC, bZIP, ERF, and HSF families, previously associated with abiotic and biotic stress response in *Solanaceae* [11,17], were found within the QTL regions, some of them with maximum root expression (Table S6). Among them, WRKY transcription factors 2 (Solyc04g078550), 5 (Solyc10g007970), 11 (Solyc08g006320), and 17 (Solyc07g051840), previously related to the drought stress response were found within clusters V, XI, VIII, and VII, respectively. Since the MAPK cascade is known to participate in the drought stress response [18,19,20], the location of the tomato MAPKs in the QTL regions (and QTL clusters) was also investigated (Table S7). As expected from the GO term enrichment analysis (Supplementary Figure S6), there were many (8) MAPKs within the QTL cluster VII, one of them, *SlMAPKKK54*, mutated at the *lycopersicum* allele (Table 3 and Table S7). Interestingly, the increasing allele at both QTLs from this cluster (Mg_WD_7 and Mn_WD_7) comes from *S. pimpinellifolium* (Table 2).

## 4. Discussion

### 4.1. S. pimpinellifolium Provides Water Deficit Tolerance Genes for Tomato Rootstocks

Around 25% of the RILs derived from *S. pimpinellifolium* conferred higher ShDW under water stress (drought tolerance) than controls, and 30% improved shoot water content when changing from control to water deficit condition (dShWC in Figure S1).

Correlation and principal component analyses (Figure 2) revealed that ShDW, ShFW, LFW, LDW, and FlN were associated under both irrigation regimes. This association was genetically supported by linkage of QTLs controlling these traits, or by the action of pleiotropic QTLs within 4 genomic regions: III, V, IX, and X. Note that the increasing allele (the allele increasing the trait mean) at those QTLs (except for FlN_WD_4.2 within cluster V) was from *S. lycopersicum*, however, due to the epistatic interactions detected for ShDW and ShWC under water deficit, the best (increasing) genotype is conditioned to the presence of a *S. pimpinellifolium* allele at a second locus (Figure S2). In the case of ShDW_WD_3 (cluster III), this locus at SNP 1495 corresponds to a previously reported QTL for iron concentrations in leaf and fruit under low iron availability (Fe_F/L_12 in [50]), in agreement with the known effect of drought on plant nutrient acquisition [27,38]. These results suggest the importance of considering epistatic interactions regarding marker-assisted selection when using wild germplasm.

Since this same RIL population was used for the genetic analysis of rootstock effects on scion traits such as total fruit weight (TFW) and fruit number (FN) under moderate salinity [46], the position of QTLs detected in both experiments can be easily compared. Thus, ShWC_WD_3 and ShWp_WD_6, were located near to rootstock QTLs controlling fruit soluble solids content under salinity, and ShWp_WD_1 close to the salt tolerance QTL in terms of commercial fruit yield (fruits heavier than 5 g, TFW > 5). Besides, QTLs for non-commercial fruit yield (fruits lighter than 5 g) under moderate salinity in chromosomes 3 (FN < 5 and TFW < 5), 6 (TFW < 5) and 11 (FN < 5) were located close to ShWC_WD_3, ShWp_WD_6 and Na_WD_11, respectively. These results and the clustering of QTLs in region X (Table S4) suggest that the ability of tomato rootstock to maintain plant water status is an important factor involved in drought and salinity tolerance. Interestingly, two aquaporin PIP2-1 coding genes were located within the drought tolerance QTL cluster IX (Table S5). On the other hand, results on positional candidates (Table 3 and Table S5) and gene enrichment analyses (Figure S3) suggest that stomatal development and closure occurred through root-to-shoot peptide signalling (clusters VIII, X, and V) could play an important role in maintaining plant water status. Furthermore, sulfate can induce stomatal closure (in [13]) and xylem [S] QTLs were in QTL clusters VI and VIII (Table 2 and Table S4). A high-affinity sulfate transporter coding gene (Solyc06g084140) mutated at the pimpinellifolium allele is a likely candidate underlying S_WD_6 at cluster VI (Table 3).

We attempted to determine genomic overlapping between the present *S. pimpinellifolium* drought tolerance regions and previously reported drought tolerance QTLs in tomato and other wild related species. The bZIP transcription factor Solyc04g078840 (Table S6), among other candidates in cluster V (including ShWC_C_4 and ShWC_WD_4) was previously found as a candidate for an interactive QTL governing stem diameter between watering regimes in tomato [31]. The *S. pennellii* introgressed regions of IL8-3 and IL2-1 conferring drought tolerance as rootstocks [33] corresponded to our genomic region between FlN_WD_8 and FlN_C_8 (two QTLs with opposite gene effects on flower number, Table 2), and cluster II (between ABA_WD_2.1 and ABA_WD_2.2), respectively. Regarding *S. habrochaites*, shoot turgor maintenance QTL under root chilling (stm9 in [34] and [35]) locates within the drought tolerance cluster IX, with the *R2R3MYB22* (Solyc09g008390, Table S6) candidate gene in common [35].

### 4.2. Root Acquisition and Long-Distance Transport of Nutrients.

Very few QTLs were detected for the xylem concentration of nutrients in well-watered plants (Table 2): 3 for B, 1 for Mg, 1 for Na, and 2 for Sr. Except for that of Na (Na_C_7) at the same position as Na_WD_7, which must correspond to the Na transporter HKT1 (as found in previous studies using this RIL population; [46,50,51]), no other was detected under water stress. Interestingly, xylem concentrations of some nutrients (Mn, Mg, Ca, Sr, Zn, P, and S) were associated under both watering regimes as visualized in the principal component analysis (Figure 2, Table S3), but the clustering of QTLs controlling these traits (QTL clusters II, IV, VI, VII, VIII, and XI in Table S4), genetically supporting such association, was only detected under water deficit. This suggests a common pathway for the acquisition and long-distance transport of these nutrients but, in well-irrigated plants, rootstock genetic composition has little phenotypic effects. In contrast, genotypic differences at those QTLs become significant under water deficit. A relationship between water deficit and nutritional stress has been often reported in the plant response to drought [26,27].

Comparing the QTLs in the present study with the 8 QTLs that determined the ability of the root to be colonized by arbuscular mycorrhizal fungi (AMF) using the same RIL population [52] showed that four of them (AMF_Col_10, AMF_Col_4, AMF_Col_6, and AMF_Col_9) were close to QTL clusters XI, IV, VI, and IX, determining xylem nutrient (especially Mn) concentration, under water deficit (Table S4). Interestingly, the plant-AMF interaction benefits plant water and nutrient acquisition [53]. The genetic control of xylem [Mn] under water deficit involved four epistatic interactions: 3 of them with Mn_WD_7 from cluster VII (Figure S2), which is particularly rich in the MAPK cascade involved in cell wall organization (Figure S3) and included *SlMAPKKK54* with a frameshift mutation at the lycopersicum allele (Table 3 and Table S7). Segregation for this mutation could explain the epistatic interactions involving Mn_WD_7, since only the pimpinellifolium allele would be functional here. Interestingly, Mn_WD_9 (linked to AMF_Col_9) and containing *ctr3* (Table S7) is also involved in 2 epistatic interactions governing xylem [Mn]. Also governing xylem [Mn] through epistasis are QTL cluster VI (linked to AMF_Col_6), which includes *SlMAPKKK42*, and Mn_WD_4 from cluster IV, linked to AMF_Col_4, which includes *SlMAPKKK32*. These two genes showed no segregation for frameshift mutations (Table S7), contrary to Cytochrome P450 86A1 (Solyc06g076800), a candidate gene within cluster VI, involved in suberin biosynthesis and mutated at the pimpinellifolium allele (Table 3). Additionally, cluster IV included a gene coding for a glycerol-phosphate acyltransferase, mutated at the pimpinellifolium allele and involved in the formation of extracellular cutin and suberin. However, the increasing allele at both QTL cluster IV and AMF_Col_4 is from *S. pimpinellifolium*. Only two candidate genes showed a frameshift mutation at the lycopersicum allele, the universal stress protein PHOS32 (Solyc04g014600), and a bZIP transcription factor (Solyc04g011670, Table 3). These results suggest a genetic relationship between the plant’s ability to be colonized by AMF and xylem concentration of certain nutrients, particularly Mn, under water deficit. This relationship would be supported by genes coding for the MAPK cascade involved in cell wall organization, suberin synthesis enzymes, and others involved in the plant response to nutrient starvation.

### 4.3. Root-to-Shoot Hormone and Peptide Signalling

Genetic regulation of xylem sap phytohormones was water regime dependent. Thus, QTLs for JA and tZ concentrations were only detected in well-watered plants, while QTLs for ABA and ZR concentrations arose under water-stress. ABA and ZR QTLs physically overlapped in one genomic region (cluster XII including ABA_WD_5 and ZR_WD_5). Another genetic connection between ABA and cytokinin signaling was found in ABA_WD_11, where in addition to genes coding for abcisic-aldehyde oxidases (Table 3), there were a sensor histidine kinase (Solyc11g071630) and a histidine phosphotransfer protein (Solyc11g070150). Only ZR QTLs overlapped with water deficit tolerance QTLs (Table 2 and Table S4), such as FlN_WD_4.2 (cluster V) and FlN_WD_9.1 (IX), with opposite gene effects, and nutrient QTLs Mn_WD_11 (XI) and Mn_WD_9 (IX), with gene effects in the same direction. A reasonable interpretation is that cytokinin ZR is important in root-to-shoot signaling of water deficit, increasing the nutrient (Mn) transport in the xylem but reducing the number of flowers (or delaying flowering). Previous studies have suggested that endogenous cytokinin play a role in conferring drought tolerance [15]. An explanation of the connection between xylem ZR and drought tolerance could be related to the role of cytokinin in the regulation of canopy senescence [39,54], a kind of stay-green trait.

Our results provide no genetic evidence that the ethylene precursor ACC acted as a root-to-shoot signal. However, the drought tolerance clusters IX and, particularly, X presented numerous genes related to the ethylene synthesis and signaling (Table S5), in addition to the ABA transporter coding gene Solyc06g091660 [55].

Plant peptides have emerged as key regulators of stress responses and tolerance [56,57], with a subtilisin-like protease, phytaspase 2, generating the peptide hormone phytosulfokine that regulates drought-induced flower drop in tomato plants [28]. The phytaspase 2 coding gene, Solyc04g078740, segregating for an SNP in the RIL population, was found as a candidate gene (and at the QTL peak) for both FlN_C_4 and FlN_WD_4.1 (Table 2 and Table S5). Two QTLs for the number of flowers under high temperatures fln4.1_T2_2E and fln4.1_T3_2E were reported in a similar genetic position using a different RIL population [58]. Other genes related to the peptide signaling pathway were found within QTL clusters III, VIII, X, and V (Table 3 and Table S5) and cytokinin QTLs ZR_WD_4 (Solyc04g077170), and ZR_WD_10 (Solyc10g011830).

### 4.4. Transcription Factors as Candidate Genes at QTLs for Water-Shortage Tolerance

Since transcription factors participate in activating/repressing gene expression in response to biotic and abiotic stresses, they have been the target of many studies to improve plant stress tolerance [11,17] through reverse genetics. Complementing this strategy, we have found transcription factors belonging to the main families previously associated with the plant stress response (DoF, WRKY, MYB, NAC, bZIP, ERF, ARF, and HSR) in the QTL clusters (Table S6). Interestingly, some of them show maximum expression in the root of Heinz, and five showed segregation for frameshift mutations: WRKY27 in the drought tolerance cluster III, R2R3MYB77 in cluster V, two MYBs within ZR_WD_9 (one of them in cluster IX), and ERF-F-5 in cluster XI.

In conclusion, around 25% of the *S. pimpinellifolium* derived RILs and at least four genomic regions could be relevant to rootstock-mediated crop improvement under water shortage. These regions corresponded to QTL clusters III (chromosome 3), V (chromosome 4), and IX and X in chromosome 9. Regions III and V were enriched in genes involved in the cell wall (both clusters), root development (III), and stomatal complex morphogenesis (V). Candidates related to the osmotic and hydraulic adjustments were found within III and IX, respectively. Transcription factors associated with the stress response and genes related to several phytohormone signaling pathways were found in all of them. Peptide signaling related genes were in regions III, V, and X, and components of the MAPK cascade were in regions V and IX. Therefore, natural genetic variability from wild *S. pimpinellifolium* that confers rootstock-mediated drought tolerance could be multigenic and distributed in a few groups of linked genes. Each group, or complex of co-adapted genes, would segregate in the RIL population, explaining each drought tolerance QTL cluster. This hypothesis would be meaningful in the context of plant evolution through adaptation, and useful to explore plant genetic resources for improving drought tolerance in tomato.

## Figures and Tables

**Table 1:** The mean and standard error of the phenotypic values observed for the analyzed traits in control lines (Bol and Bol/Bol) for well-watered (_C) and water-stressed (_D) plants. Minimum (Mi) and maximum (Ma) means in RIL population, estimated broad-sense heritabilities (H^2^) and *p*-values of the effects (genotype, G; treatment, E; and GxE interaction) in the mixed model analysis are also included.

Abbreviation	Trait	Bol/Bol_C	Bol_C	Bol/Bol_D	Bol_D	Mi_C	Ma_C	Mi_D	Ma_D	H2_C	H2_D	G	E	G × E
ABA	Xylem sap [ABA]	17.91 ± 6.2	8.48 ± 4.19	22.76 ± 8.94	8.3 ± 2.12	–0.85	36.96	–0.56	28.03	0.1505	0.1544	<0.0001	0.1815	0.0932
ACC	Xylem sap [ACC]	9 ± 4.5	8.96 ± 4.48	4.66 ± 2.33	7.97 ± 3.99	0.00	23.32	0.00	26.93	0.0308	0.0724	0.0396	0.8182	0.3208
As	Xylem sap [As] (mg/L)	0.11 ± 0.03	0.12 ± 0.03	0.1 ± 0.01	0.15 ± 0.04	0.01	0.31	0.01	0.24	0.0000	0.1325	0.2856	0.7236	0.2377
B	Xylem sap [B] (mg/L)	0.32 ± 0.1	0.2 ± 0.02	0.25 ± 0.03	0.36 ± 0.09	–0.06	0.83	–0.02	0.81	0.3007	0.3249	<0.0001	0.1699	<0.0001
Ca	Xylem sap [Ca] (mg/L)	154.62 ± 23.41	85.25 ± 9.83	170.87 ± 44.94	269.57 ± 27.46	62.35	549.20	56.06	619.80	0.1539	0.2800	0.0021	0.3211	<0.0001
Cr	Xylem sap [Cr] (mg/L)	0.03 ± 0.02	0.01 ± 0.01	0 ± 0	0 ± 0	0.00	0.14	–0.01	0.09	0.0670	0.1894	0.0475	0.658	0.016
Cu	Xylem sap [Cu] (mg/L)	0.1 ± 0.01	0.08 ± 0.03	0.08 ± 0	0.12 ± 0.0033	0.04	0.33	0.04	0.43	0.1859	0.1218	0.0003	0.9083	0.1081
Fe	Xylem sap [Fe] (mg/L)	0.36 ± 0.2	0.19 ± 0.02	0.25 ± 0.08	0.37 ± 0.12	0.04	1.61	0.09	1.46	0.1711	0.2052	<0.0001	0.997	0.2186
FlN	Number of flowers	5.67 ± 0.88	3.67 ± 0.33	4.33 ± 0.88	3.67 ± 0.88	2.69	9.40	1.54	7.04	0.0214	0.1638	<0.0001	0.7691	0.5706
iP	Xylem sap [isopentenyladenine]	2.81 ± 0.33	2.43 ± 0.11	2.14 ± 0.16	2.01 ± 0.13	–0.05	20.54	0.00	11.09	0.1316	0.0905	<0.0001	0.2615	0.0237
JA	Xylem sap [Jasmonic acid]	111.67 ± 49.84	44.6 ± 42.45	67.79 ± 42.62	19.89 ± 11.72	0.00	137.08	–3.03	166.23	0.1461	0.1970	0.0004	0.1956	0.0045
K	Xylem sap [K] (mg/L)	522.65 ± 104.91	398.33 ± 23.16	580.38 ± 140.63	622.03 ± 46.16	242.79	897.09	184.37	1053.70	0.0557	0.1324	0.0692	0.8391	0.0595
LA	Area of fifth leaf (cm2)	1052.67 ± 11.34	898.1 ± 73.44	594.13 ± 68.82	829.87 ± 79.35	563.44	1260.69	430.09	1194.51	0.1708	0.1189	0.0003	0.0685	0.013
LDW	Leaf Dry Weight (g)	66.79 ± 8.24	39.46 ± 0.82	47.69 ± 1.2	42.84 ± 1.27	22.64	97.41	23.37	89.95	0.0496	0.3147	<0.0001	0.6744	0.1598
LFW	Leaf Fresh Weight (g)	666.67 ± 70.07	486.67 ± 38.75	561.67 ± 7.88	478 ± 28.38	235.44	1118.12	213.33	994.00	0.2912	0.4482	<0.0001	0.1812	0.0004
Li	Xylem sap [Li] (mg/L)	0.09 ± 0.09	0.05 ± 0.05	0.18 ± 0.06	0.19 ± 0.05	0.00	0.18	0.00	0.32	0.0000	0.2978	0.0005	0.0093	0.0003
Mg	Xylem sap [Mg] (mg/L)	39.87 ± 4.24	20.44 ± 3.51	48.91 ± 13.91	62.56 ± 6.4	15.03	120.00	14.90	158.58	0.1763	0.2142	0.0001	0.5589	0.0015
Mn	Xylem sap [Mn] (mg/L)	0.68 ± 0.13	0.39 ± 0.04	0.73 ± 0.25	1.14 ± 0.12	0.26	3.94	0.25	3.26	0.1946	0.2906	0.0003	0.923	<0.0001
Mo	Xylem sap [Mo] (mg/L)	0.01 ± 0.0033	0.02 ± 0.01	0.01 ± 0.01	0.02 ± 0.01	0.00	0.36	0.00	0.27	0.0773	0.1752	0.0294	0.5448	0.0466
Na	Xylem sap [Na] (mg/L)	4.04 ± 1.39	2.43 ± 0.39	4.51 ± 2.26	3.33 ± 2.73	1.52	34.14	0.71	29.21	0.3642	0.1961	<0.0001	0.8547	0.1227
P	Xylem sap [P]	30.82 ± 9.56	23.76 ± 4.69	30.77 ± 9.13	42.27 ± 7.99	11.51	81.16	5.71	92.95	0.1535	0.2151	0.0001	0.314	0.0017
S	Xylem sap [S] (mg/L)	40.6 ± 8.49	24.4 ± 3.33	57.22 ± 19.81	75.52 ± 20.7	16.04	137.62	2.97	183.80	0.1971	0.2894	<0.0001	0.5098	<0.0001
Sb	Xylem sap [Sb] (mg/L)	0.01 ± 0.01	0 ± 0	0.0033 ± 0.0033	0 ± 0	0.00	0.15	–0.01	0.11	0.0000	0.1722	0.0866	0.5837	0.4646
Se	Xylem sap [Se] (mg/L)	0.04 ± 0.04	0.04 ± 0.03	0.03 ± 0.02	0.01 ± 0.01	0.00	0.57	0.00	0.13	0.1869	0.0594	<0.0001	0.0134	0.0011
ShDW	Total Shoot Dry Weight (g)	117.47 ± 13.9	68.13 ± 4.45	89.67 ± 2.69	74.83 ± 1.53	44.90	164.19	44.01	141.53	0.1299	0.3449	<0.0001	0.4472	0.1096
ShFW	Total Shoot Fresh Weight (g)	1203 ± 125.05	849.67 ± 90.71	1056.67 ± 5.93	860 ± 46.36	430.58	2087.98	414.00	1669.82	0.2858	0.4469	<0.0001	0.1178	0.0003
ShWC	Shoot water content (g)	1085.55 ± 111.74	781.54 ± 86.32	967.01 ± 5.73	785.17 ± 47.32	385.96	1923.97	369.91	1536.15	0.2975	0.4492	<0.0001	0.1063	0.0002
ShWp	Shoot water proportion	0.9 ± 0.0033	0.92 ± 0.0033	0.91 ± 0.0033	0.91 ± 0.0033	0.89	0.93	0.88	0.93	0.2000	0.2408	<0.0001	0.024	0.1937
SPAD	SPAD readings	47.23 ± 0.54	44.83 ± 0.88	45.67 ± 0.38	43.77 ± 0.87	39.00	51.80	31.97	50.73	0.6843	0.3698	<0.0001	0.007	<0.0001
Sr	Xylem sap [Sr] (mg/L)	0.33 ± 0.04	0.21 ± 0.04	0.43 ± 0.12	0.65 ± 0.05	0.14	1.31	0.13	1.53	0.1944	0.2579	0.0003	0.8104	<0.0001
tZ	Xylem sap [trans–Zeatin]	17.02 ± 3.33	9.88 ± 2.35	13.97 ± 2.42	8.34 ± 2	5.17	29.39	5.07	32.72	0.1809	0.0565	0.0006	0.5577	0.0059
Zn	Xylem sap [Zn] (mg/L)	0.46 ± 0.06	0.28 ± 0.06	0.42 ± 0.09	0.57 ± 0.03	0.15	1.03	0.17	1.47	0.1510	0.1742	0.0004	0.3494	0.0172
ZR	Xylem sap [trans–Zeatin Riboside]	12.6 ± 0.82	9.88 ± 0.18	8.59 ± 1.13	2.28 ± 2.28	0.00	12.34	0.11	12.56	0.1039	0.1369	0.0108	0.0888	0.0162

**Table 2:** List of QTLs (named by the trait, the irrigation regime: C or WD, and the chromosome) that were detected by using MQM procedure (5% overall significance level) and corresponding SNPs (mostly SolCAP SNPs named by the number) at the LOD peak. The map position (cM) of QTL peaks in the tomato chromosomes (Chr) are indicated. The estimated additive value is a (negative when the allele increasing the trait mean comes from *S. pimpinellifolium*), its percentage of explained variance, PEV, and the physical genomic region where several closely linked QTLs are present, forming a cluster (Cluster), are included. Cluster between parenthesis indicates QTL showing weaker linkage with the cluster. For QTLs included within each genomic region, see also Table S4.

Cluster	QTL	Chr.	Position	SNP	LOD	PEV	a
	ABA_WD_11	11	75.154	56,353–32,118	2.04	5.8	0.97
	ABA_WD_2.1	2	8.856	36,400	3.64	10.7	1.39
	ABA_WD_2.2	2	76.525	29,914	2.91	8.5	−1.14
XII	ABA_WD_5	5	108.514	339–354	2.9	8.4	−1.15
	B_C_3	3	64.335	35,397–35,459	2.48	6.1	0.04
	B_C_5	5	62.107	51,061	2.12	5.2	−0.04
	B_C_6	6	10.733	65,686	2.3	5.7	0.04
VI	B_WD_6	6	96.06	C2_At1g20050_230_b	3.44	11.4	0.05
	B_WD_9	9	115.383	63,663	2.44	7.9	0.04
IV	Ca_WD_4	4	28.574	41,577	2.82	7.5	−33.11
VI	Ca_WD_6	6	96.06	C2_At1g20050_230_b	2.43	6.4	29.42
VIII	Ca_WD_8	8	11.65	56,575	3.48	9.4	35.43
	Cu_WD_1	1	42.436	59,885	5.67	19.4	0.04
	FlN_C_4	4	39.43	17,956	5.01	13.4	0.46
	FlN_C_5	5	60.926	CL017527-0194	3.04	7.8	0.34
	FlN_C_6	6	0.182	26,804	2.63	6.7	−0.31
	FlN_C_8	8	102.434	58,992	2.1	5.3	0.27
I	FlN_WD_1	1	47.252	51,462	2.97	4.9	0.26
	FlN_WD_11	11	40.373	9508	2.36	3.8	0.23
III	FlN_WD_3	3	52.869	30,678	3.22	5.3	0.26
	FlN_WD_4.1	4	39.43	17,956	4.14	6.9	0.49
V	FlN_WD_4.2	4	49.35	47,259	2.42	3.9	−0.35
	FlN_WD_5	5	32.533	23,804	4.82	8.2	0.33
	FlN_WD_8	8	86.586	65,161–65,188	2.37	3.5	−1.67
IX	FlN_WD_9.1	9	18.634	57,922–57,900	2.72	4.4	0.25
X	FlN_WD_9.2	9	93.838	36,845–69,503	2.59	4.2	0.25
I	JA_C_1	1	50.094	50,470	2.41	6.5	7.11
	JA_C_7	7	30.461	68,008	2.14	5.7	−6.39
	LA_C_11	11	24.632	66,141	2.25	6.4	32.18
	LA_C_4	4	3.085	21,317	2.74	7.8	−36.11
	LA_C_6	6	101.994	54,183	3.24	9.3	−39.51
V	LDW_C_4	4	59.661	47,590	2.51	8.5	3.14
IX	LDW_WD_9	9	21.4	39,886	2.32	7.6	3.44
V	LFW_C_4	4	56.517	47,487	2.79	9.3	39.27
X	LFW_WD_9	9	94.89	69,503	2.45	8.7	37.52
	Mg_C_3	3	29.052	55,002	2.26	8.1	6.72
XI	Mg_WD_10	10	22.598	17,838	2.24	5.7	8.14
II	Mg_WD_2	2	46.39	T0562-49,497	2.5	6.4	8.41
IV	Mg_WD_4	4	26.383	16,978	2.15	5.5	−7.79
VI	Mg_WD_6	6	96.06	C2_At1g20050_230_b	2.88	7.4	8.97
VII	Mg_WD_7	7	68.714	53,534	2.52	6.5	−8.48
XI	Mn_WD_10	10	22.598	17,838	3.61	8.8	0.19
II	Mn_WD_2	2	46.39	T0562-49,497	3.13	7.5	0.17
IV	Mn_WD_4	4	26.174	41,648–41,623	2.65	6.3	−0.16
VII	Mn_WD_7	7	68.714	53,534	2.56	6.1	−0.16
IX	Mn_WD_9	9	19.634	57,922–57,900	2.62	6.2	−0.16
	Na_C_7	7	38.818	67,869	12.26	36.8	−3.49
	Na_WD_11	11	89.544	56,158	4.09	11.4	2.08
	Na_WD_7	7	38.818	67,869	6.23	18.2	−2.65
XI	P_WD_10	10	24.053	46,416	2.76	7.8	5.12
VI	P_WD_6	6	95.563	2819_5_183_b	4.06	11.8	6.43
VI	S_WD_6	6	96.06	C2_At1g20050_230_b	3.86	10.2	13.00
VIII	S_WD_8	8	11.65	56,575	2.2	5.6	9.46
V	ShDW_C_4	4	56.517	47,487	2.5	8.6	5.19
III	ShDW_DW_3	3	52.651	30,704	3.16	9	5.55
V	ShFW_C_4	4	56.517	47,487	2.39	8.4	68.79
X	ShFW_WD_9	9	83.507	58,234	2.41	8.6	61.68
V	ShWC_C_4	4	56.517	47,487	3.05	9.8	68.25
	ShWC_WD_1	1	12.421	60,303	2.33	6	−49.63
	ShWC_WD_3	3	91.561	62,473	2.26	5.7	−52.09
V	ShWC_WD_4	4	63.868	3872	2.81	7.3	53.67
X	ShWC_WD_9	9	94.89	69,503	3.94	10.4	64.86
	ShWp_C_12	12	88.198	32,743	2.9	7.1	0.00
	ShWp_C_3.1	3	4.081	63,211	4.17	10.4	0.00
	ShWp_C_3.2	3	90.075	62,473	2.4	5.8	0.00
	ShWp_C_9	9	103.792	69,686–63,663	3.22	7.9	0.00
	ShWp_WD_1	1	78.318	27,588–27,600	2.76	6.8	0.00
	ShWp_WD_6.1	6	51.444	27,197	2.16	5.2	0.00
	ShWp_WD_6.2	6	60.181	42,119	6.5	17.2	−0.01
	ShWp_WD_6.3	6	74.588	57,435	2.91	7.2	0.00
X	ShWp_WD_9	9	84.784	58,253	2.97	7.3	0.00
	Sr_C_1	1	152.18	SGN-U313729_snp305	3.58	10.8	−0.25
	Sr_C_9	9	32.383	39,735	2.43	7.2	0.08
IV	Sr_WD_4	4	26.383	16,978	2.94	7.7	−0.08
VI	Sr_WD_6	6	96.06	C2_At1g20050_230_b	3.11	8.1	0.08
VIII	Sr_WD_8	8	11.65	56,575	2.31	5.9	0.07
	tZ_C_11	11	52.492	2966	2.3	8.3	−1.45
II	Zn_WD_2	2	46.39	T0562-49,497	2.69	6.8	0.07
VI	Zn_WD_6	6	96.06	C2_At1g20050_230_b	2.94	7.4	0.16
(XI)	ZR_WD_10	10	45.374	52,018	3.42	9.3	0.86
V	ZR_WD_4	4	48.186	47,229	2.42	5.9	0.89
XII	ZR_WD_5	5	110.72	37,819	2.31	6.2	−0.72
(IX)	ZR_WD_9	9	2.545	14,673–58,096	2.81	7.6	−0.83

**Table 3:** Summary list of candidate genes in cluster regions and QTLs for compounds in the xylem sap, and some segregating for frameshift Indels [48] in parental genomes, E9 or L5, (Mut). The mRNA reference, its starting physical position in the chromosome (Start), its relative root expression (Exp) in Heinz cultivar (Max: maximum, H: high, M: medium, VL: very low, L: low and N: no data), and the number of genes counted from the QTL peak (Ord) are shown.

Cluster/QTL	Mut	Exp	Start	Annotation	mRNA	Ord
Sr_C_1		M	97,531,670	Magnesium transporter NIPA2	Solyc01g111190.2.1	6
Sr_C_9		N	3,902,289	Cation/H+ antiporter	Solyc09g010530.2.1	53
VIII		L	876,173	ChaC cation transport regulator-like 1	Solyc08g006150.2.1	38
VIII		Max	1,096,757	Glutamate-gated kainate-type ion channel receptor	Solyc08g006500.2.1	4
VIII	L5	Max	2,169,906	Subtilisin-like protease (4 copies in tandem)	Solyc08g007670.1.1	113
VIII		Max	2,541,278	Aquaporin (aquaporin PIP-type pTOM75)	Solyc08g008050.2.1	149
VIII	L5	VL	2,654,348	Cation/H(+) antiporter 18	Solyc08g008190.2.1	162
VIII	L5	N	2,658,313	Cation/H(+) antiporter 18	Solyc08g008200.1.1	163
VI		H	47,496,433	Zinc-transporter-like protein	Solyc06g076440.1.1	151
VI	L5	Max	47,705,394	Cytochrome P450 86A1	Solyc06g076800.2.1	117
VI	L5	Max	48,070,379	SAGA-associated factor 11 homolog	Solyc06g082160.2.1	80
VI		Med	48,142,943	Cation/H(+) antiporter 14	Solyc06g082230.2.1	73
VI	L5	Max	48,687,538	E3 ubiquitin-protein ligase CHIP	Solyc06g083150.2.1	17
VI	L5	Max	49,364,970	High affinity sulfate transporter 1	Solyc06g084140.2.1	116
IV	L5	Max	4,029,691	ER glycerol-phosphate acyltransferase	Solyc04g011600.2.1	110
IV	E9	Max	4,150,679	BZIP transcription factor	Solyc04g011670.2.1	103
IV	L5	N	4,226,837	Glutaredoxin (2 copies)	Solyc04g011780.1.1	92
IV		Max	4,376,146	Ethylene responsive transcription factor 2a (ABR1-like)	Solyc04g012050.2.1	66
IV	E9	VL	4,872,992	Universal stress protein (PHOS32)	Solyc04g014600.2.1	9
IV		M	5,226,676	Heavy metal transport/detoxification protein (2 copies)	Solyc04g015020.2.1	0
IV	L5	N	5,133,669	Outward rectifying potassium channel	Solyc04g014880.2.1	0
IV	L5	Max	5,458,419	NRC1	Solyc04g015250.1.1	6
IV		H	5,491,465	MYB transcription factor (protein PHR1-LIKE 3)	Solyc04g015290.2.1	10
IV		VL	6,590,404	Cation/H(+) antiporter 28	Solyc04g015990.1.1	78
XI		Max	763,795	Ca-activated outward-rectifying potassium channel 1	Solyc10g006010.2.1	128
XI		Max	950,380	Purple acid phosphatase	Solyc10g006300.2.1	99
XI	L5	H	1,911,958	Organic anion transporter	Solyc10g007610.2.1	2
XI	L5	Max	1,919,334	Glutathione S-transferase (2 copies in tandem)	Solyc10g007620.1.1	3
XI		H	1,981,130	Mg2+ transporter protein CorA family protein	Solyc10g007740.2.1	15
VII	E9	M	60,468,833	MAPKKK54	Solyc07g051920.1.1	107
VII		VL	60,504,547	C4-dicarboxylate transporter/malic acid transport family	Solyc07g051950.2.1	104
VII		H	60,921,179	Glutamate-gated kainate-type ion channel receptor (2 copies)	Solyc07g052400.2.1	62
VII		Max	62,362,157	Two-pore calcium channel 2	Solyc07g053970.2.1	92
II		M	44,832,287	Copper transport protein 86 (ataxin-10)	Solyc02g080650.2.1	8
II		Max	45,468,042	Yellow stripe-like protein 2.1 (YSL7)	Solyc02g081570.2.1	100
II		M	45,492,312	Zinc transporter protein (zinc transporter 3)	Solyc02g081600.2.1	103
II		M	45,804,741	High affinity copper uptake protein (Cu transporter 5)	Solyc02g082080.1.1	151
IX	L5	N	1,176,696	MYB transcription factor (SRM1-like)	Solyc09g007580.1.1	79
IX		M	1,401,117	Mn transporter mntH (ethylene signaling protein)	Solyc09g007870.2.1	50
IX		H	1,607,740	Membrane magnesium transporter 1	Solyc09g008140.2.1	23
IX		Max	1,718,596	MYB transcription factor 38 (protein blind-like1, bli1)	Solyc09g008250.2.1	12
V	L5	Max	63,103,059	Cytochrome P450 CYP2 subfamily (3 copies in tandem)	Solyc04g078340.2.1	15
V		Max	63,259,221	WRKY transcription factor 7	Solyc04g078550.2.1	6
V	L5	Max	63,627,437	Anthocyanidin 3-O-glucosyltransferase	Solyc04g079030.2.1	54
V		Max	63,744,550	Gibberellin receptor GID1L2	Solyc04g079190.2.1	70
V	L5	M	63,888,688	MYB transcription factor 77	Solyc04g079360.1.1	87
V	L5	L	64,050,601	Cytochrome P450 CYP2 subfamily	Solyc04g079680.2.1	115
V	L5	M	64,193,977	Seryl-tRNA synthetase	Solyc04g079870.1.1	134
XII	L5	M	63,229,667	Zeatin O-β-D-xylosyltransferase	Solyc05g053120.1.1	223
XII		M	63,498,355	Zeatin O-β-D-xylosyltransferase	Solyc05g053400.1.1	195
XII		M	63,610,661	9-cis-epoxycarotenoid dioxygenase 6	Solyc05g053530.1.1	182
XII		M	64,775,972	Cysteine desulfurase, molybdenum cofactor biosynthesis	Solyc05g055000.2.1	36
XII		M	64,859,523	Ethylene receptor	Solyc05g055070.2.1	29
XII	L5	Max	64,872,981	Ubiquitin carboxyl-terminal hydrolase family	Solyc05g055090.2.1	27
XII		Max	65,269,467	Isopentenyl-diphosphate delta-isomerase	Solyc05g055760.2.1	34
XII	L5	Max	65,399,111	Serine/threonine-protein phosphatase (MAIN-LIKE 2)	Solyc05g056010.2.1	59
ZR_WD_9	L5	Max	21,705	MYB transcription factor-like	Solyc09g005030.1.1	109
ZR_WD_9		VL	1,356,808	Cytokinin riboside 5’-monophosphate phosphoribohydrolase LOG3	Solyc09g007830.2.1	70
ZR_WD_9	L5	Max	2,036,208	NCS1 family transporter	Solyc09g008550.2.1	142
ABA_WD_2.1		N	36,230,064	Short-chain dehydrogenase/reductase family protein	Solyc02g065060.2.1	46
ABA_WD_2.1	L5	N	36,835,375	Glucosyltransferase-like protein	Solyc02g065670.1.1	13
ABA_WD_2.1	E9	VL	37,817,307	Glucosyltransferase-like protein	Solyc02g067690.2.1	116
ABA_WD_11		N	54,668,129	Histidine phosphotransfer protein	Solyc11g070150.1.1	49
ABA_WD_11	L5	Max	55,006,926	Aldehyde oxidase (abscisic-aldehyde oxidase-like)	Solyc11g071580.1.1	93
ABA_WD_11	L5	Max	55,013,280	Aldehyde oxidase (abscisic-aldehyde oxidase-like)	Solyc11g071590.1.1	94
ABA_WD_11		N	55,049,892	Sensor histidine kinase LuxQ	Solyc11g071630.1.1	98

## Supplementary Materials

The following are available online at https://www.mdpi.com/2073-4425/12/1/10/s1, Figure S1: Cumulative distributions of total shoot dried weight (ShDW), and changes in ShDW (dShDW), ShFW (dShFW), ShWp (dShWp), and ShWC (dShWC). The position of Bol (black) and Bol/Bol (red) are indicated; Figure S2: Genotypic means and standard errors for significant epistatic interactions between QTL markers and/or cofactors governing ShDW_WD, ShWC_WD, B_C, Mg_WD, JA_C, and Mn_WD. Homozygotes for the lycopersicum or the pimpinellifolium allele are coded as a or b, respectively, at the first locus (X axis), and a red square (a) or a blue square (b) at the second locus; Figure S3: Overrepresented biological processes, molecular functions and cellular components within clustered QTL genomic regions using the singular enrichment analysis tool with the Fisher’s exact with FDR multiple test correction [49] at the AgriGo platform (http://systemsbiology.cau.edu.cn/agriGOv2/); Table S1: *p*-values of significantly (*p* < 0.05) different traits between controls Bol versus Bol/Bol under both watering regimes (C and WD). (+) means grafting has an increasing effect on the trait; Table S2: Pearson coefficients between significantly correlated traits (*p* ≤ 0.05) for plants under control (_C) and water deficit (_WD). In bold, for each trait between irrigation regimes; Table S3: Correlations between principal components (1 and 2) and original traits; Table S4: QTLs included within each cluster or genomic region. In parenthesis, QTLs showing a weaker linkage; Table S5: Summary list of candidate genes in drought tolerance QTL clusters, some segregating for frameshift Indels [48] in parental genomes, E9 or L5, (Mut.). The mRNA reference, its starting physical position in the chromosome (Start), its relative root expression (Exp.) in Heinz cultivar (Max, maximum; H, high; M, medium; VL, very low; L, low; and N, no data), and the number of genes counted from the QTL peak (Ord.) are also shown; Table S6: List of transcription factors belonging to the main families that have been previously associated with the plant stress response (DoF, WRKY, MYB, NAC, bZIP, ERF, ARF, and HSR) found among candidate genes in cluster regions and QTLs for phytohormones in the xylem sap, some segregating for frameshift Indels [48] in parental genomes, E9 or L5, (Mut.). The mRNA reference, its starting physical position in the chromosome (Start), and its relative root expression (Exp.) in Heinz cultivar (Max, maximum; H, high; M, medium; VL, very low; L, low; and N, no data); Table S7: List of MAPKinases found among candidate genes in cluster regions and QTLs for compounds in the xylem sap, some segregating for frameshift Indels [48] in parental genomes, E9 or L5, (Mut). The mRNA reference, its starting physical position in the chromosome (Start), and its relative root expression (Exp) in Heinz cultivar (Max, maximum; H, high; M, medium; VL, very low; L, low; and N, no data).
