# Supplementary material for: Genetic Analysis of Root-to-Shoot Signaling and Rootstock-Mediated Tolerance to Water Deficit in Tomato
Source: Genes (Basel). 2020 Dec 23;12(1):10. doi: 10.3390/genes12010010 (PMC7823420; doi:10.3390/genes12010010)
Supplement: Supplementary file 1 [file genes-12-00010-s001.pdf]

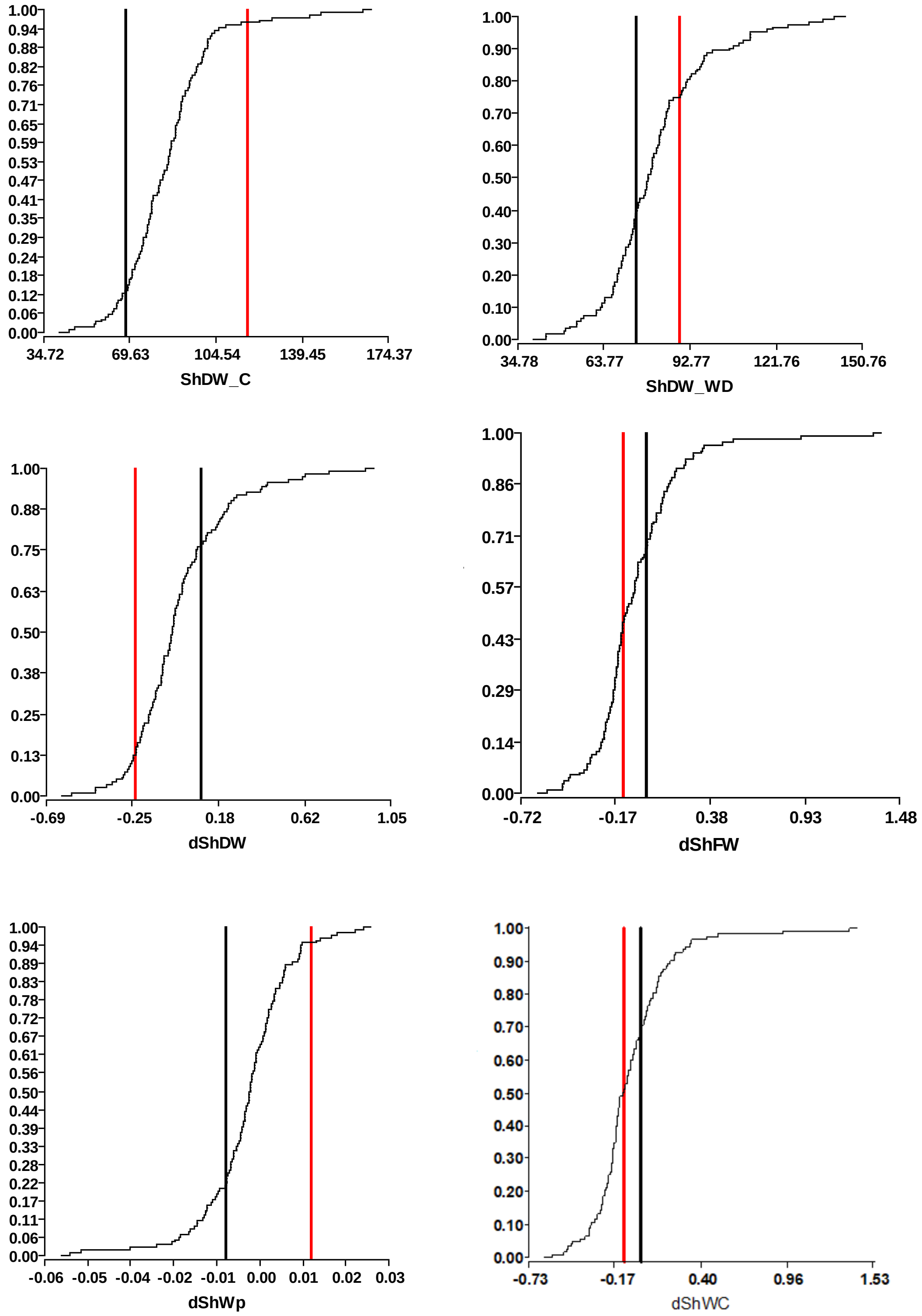

Figure S1: Cumulative distributions of total shoot dried weight (ShDW), and changes in ShDW (dShDW), ShFW (dShFW), ShWp (dShWp) and ShWC (dShWC). The position of Bol (black line) and Bol/Bol (red line) are indicated.

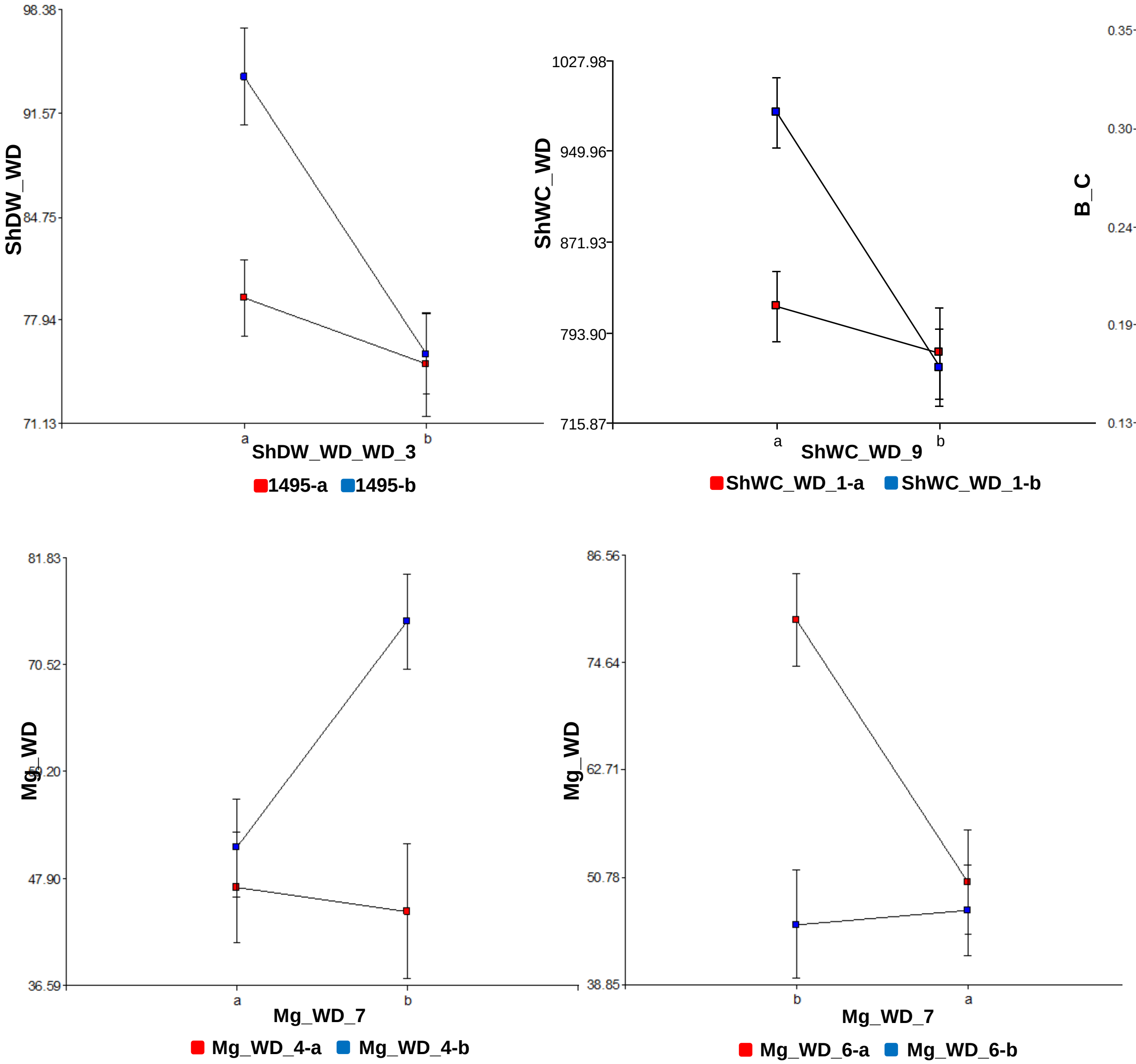

Figure S2: Genotypic means and standard errors for significant epistatic interactions between QTL markers and/or cofactors governing ShDW_WD, ShWC_WD, B_C, Mg_WD, JA_C and Mn_WD. Homozygotes for the lycopersicum or the pimpinellifolium allele are coded as **a** or **b**, respectively, at the first locus (X axis), and a red square (**a**) or a blue square (**b**) at the second locus.

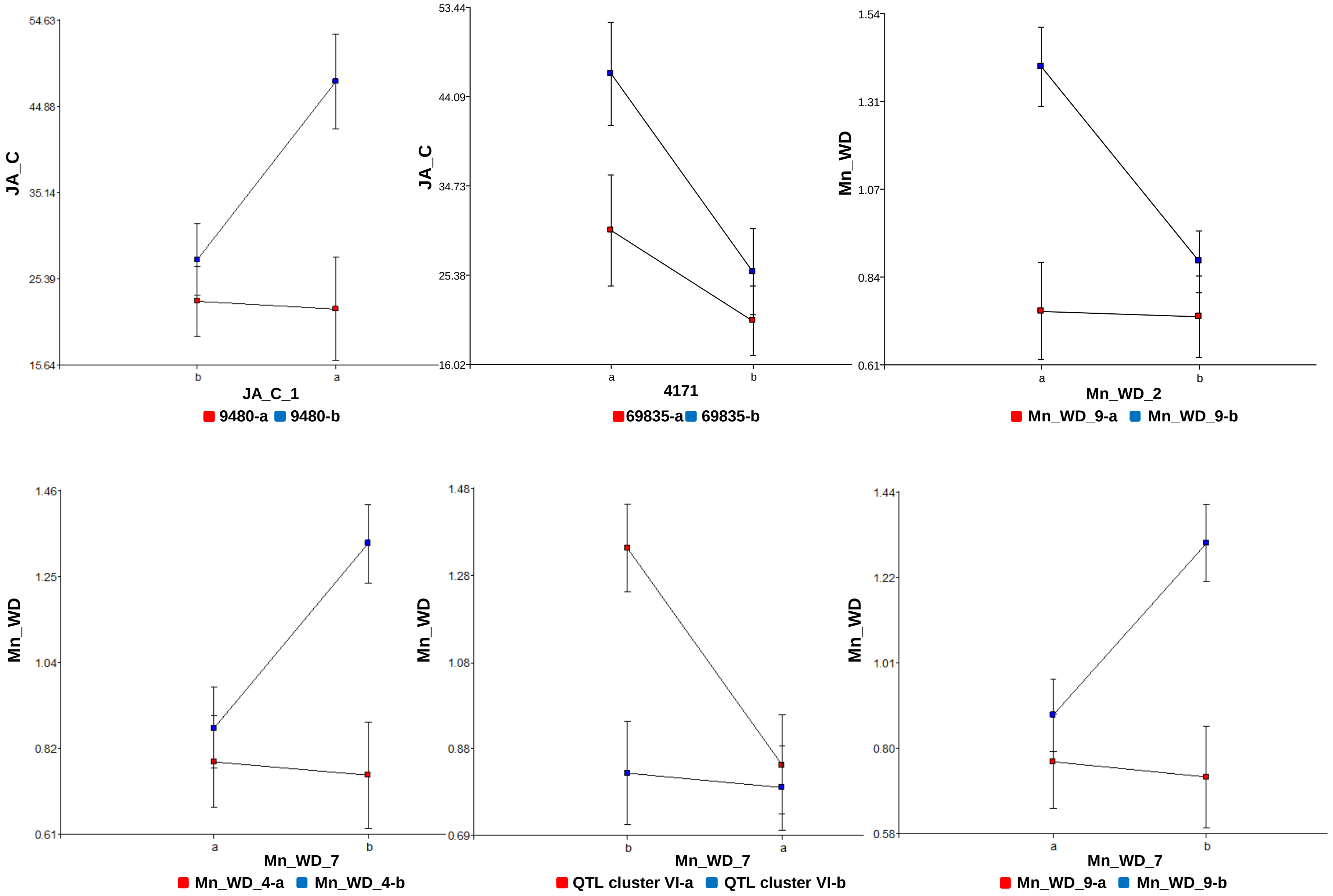
54.63
44.88
35.14
25.39
15.64
JA_C
b
a
JA_C_1
9480-a
9480-b
53.44
44.09
34.73
25.38
16.02
JA_C
a
b
4171
69835-a
69835-b
1.54
1.31
1.07
0.84
0.61
Mn_WD
a
b
Mn_WD_2
Mn_WD_9-a
Mn_WD_9-b
1.46
1.25
1.04
0.82
0.61
Mn_WD
a
b
Mn_WD_7
Mn_WD_4-a
Mn_WD_4-b
1.48
1.28
1.08
0.88
0.69
Mn_WD
b
a
Mn_WD_7
QTL cluster VI-a
QTL cluster VI-b
1.44
1.22
1.01
0.80
0.58
Mn_WD
a
b
Mn_WD_7
Mn_WD_9-a
Mn_WD_9-b

Figure S3: Overrepresented Biological Processes, Molecular Functions and Cellular Components within clustered QTL genomic regions using the Singular Enrichment Analysis tool with the Fisher´s Exact with FDR multiple test correction [49] at the AgriGo platform (http://systemsbiology.cau.edu.cn/agriGOv2/).

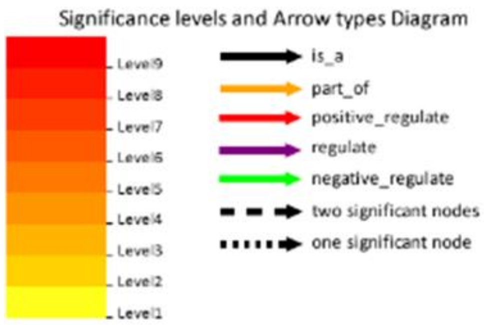

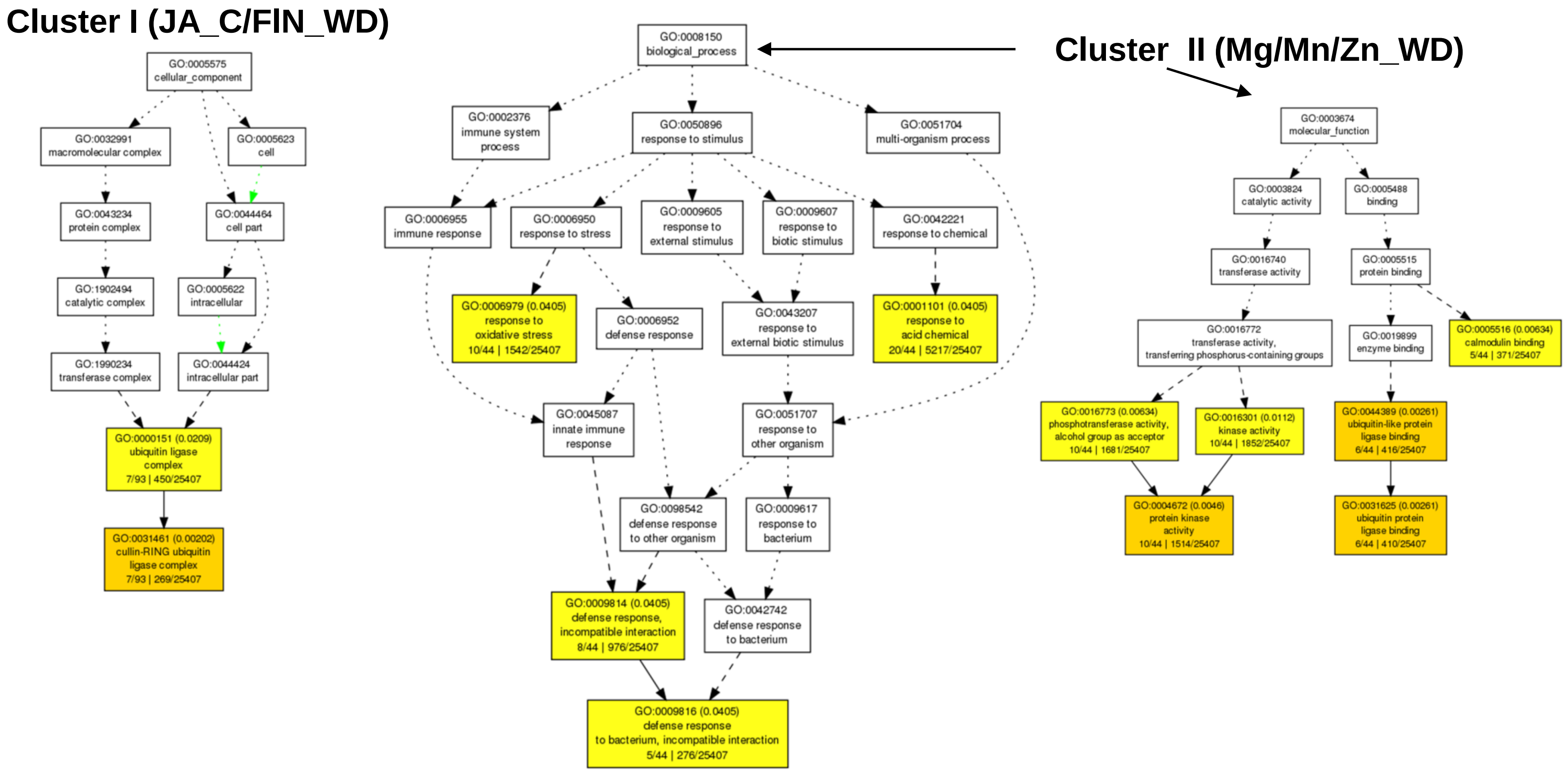
Cluster I (JA_C/FIN_WD)
GO:0005575 cellular_component
GO:0032991 macromolecular complex
GO:0005623 cell
GO:0043234 protein complex
GO:0044464 cell part
GO:1902494 catalytic complex
GO:0005622 intracellular
GO:1990234 transferase complex
GO:0044424 intracellular part
GO:0000151 (0.0209) ubiquitin ligase complex 7/93 | 450/25407
GO:0031461 (0.00202) cullin-RING ubiquitin ligase complex 7/93 | 269/25407
GO:0008150 biological_process
Cluster II (Mg/Mn/Zn_WD)
GO:0002376 immune system process
GO:0050896 response to stimulus
GO:0051704 multi-organism process
GO:0006955 immune response
GO:0006950 response to stress
GO:0009605 response to external stimulus
GO:0009607 response to biotic stimulus
GO:0042221 response to chemical
GO:0006979 (0.0405) response to oxidative stress 10/44 | 1542/25407
GO:0006952 defense response
GO:0043207 response to external biotic stimulus
GO:0001101 (0.0405) response to acid chemical 20/44 | 5217/25407
GO:0045087 innate immune response
GO:0051707 response to other organism
GO:0098542 defense response to other organism
GO:0009617 response to bacterium
GO:0009814 (0.0405) defense response, incompatible interaction 8/44 | 976/25407
GO:0042742 defense response to bacterium
GO:0009816 (0.0405) defense response to bacterium, incompatible interaction 5/44 | 276/25407
GO:0003674 molecular_function
GO:0003824 catalytic activity
GO:0005488 binding
GO:0016740 transferase activity
GO:0005515 protein binding
GO:0016772 transferase activity, transferring phosphorus-containing groups
GO:0019899 enzyme binding
GO:0005516 (0.00634) calmodulin binding 5/44 | 371/25407
GO:0016773 (0.00634) phosphotransferase activity, alcohol group as acceptor 10/44 | 1681/25407
GO:0016301 (0.0112) kinase activity 10/44 | 1852/25407
GO:0044389 (0.00261) ubiquitin-like protein ligase binding 6/44 | 416/25407
GO:0004672 (0.0046) protein kinase activity 10/44 | 1514/25407
GO:0031625 (0.00261) ubiquitin protein ligase binding 6/44 | 410/25407

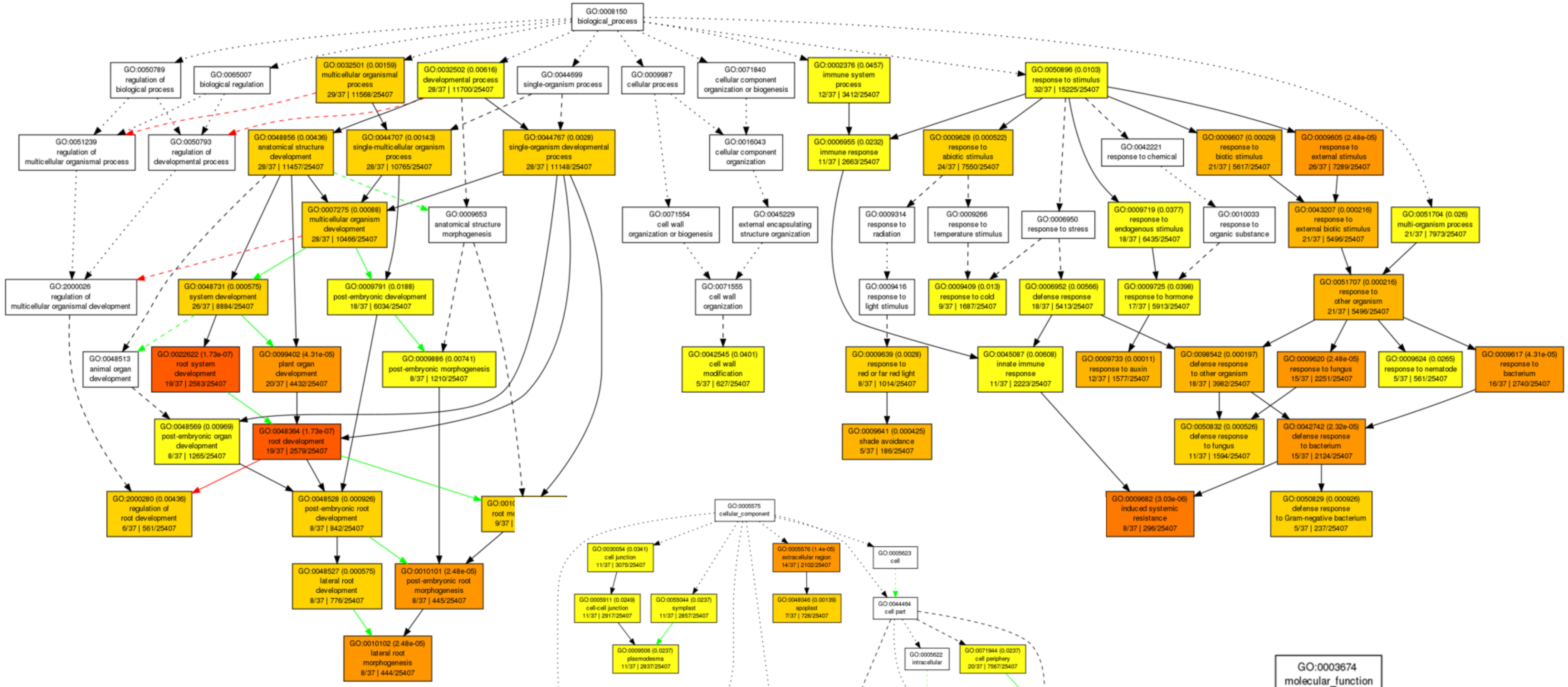

Cluster III (ShDW/FlN_WD)

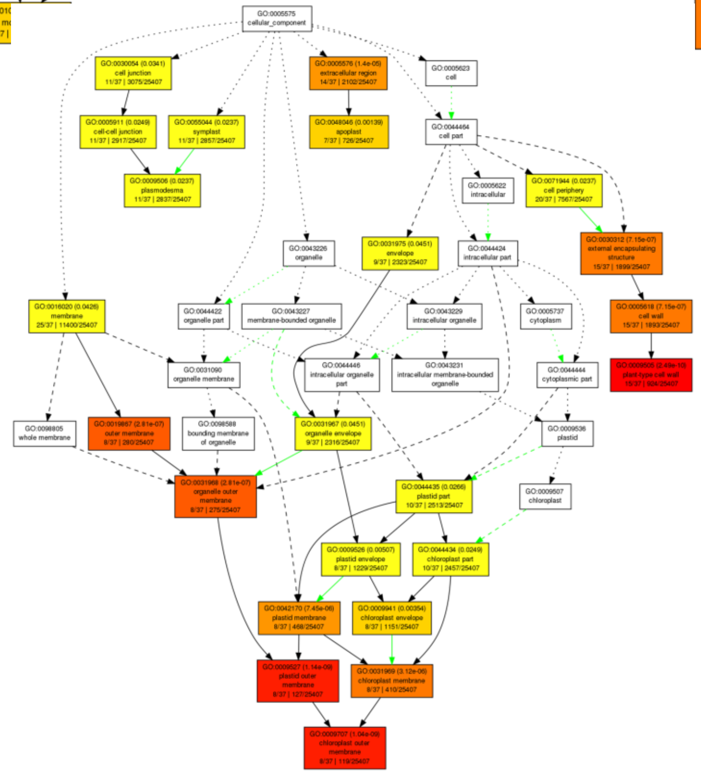

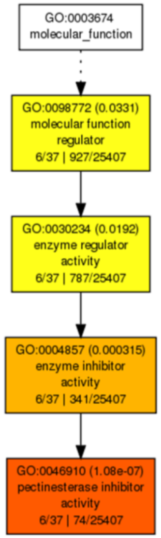

Cluster IV (Mn/Mg/Ca/Sr_WD)

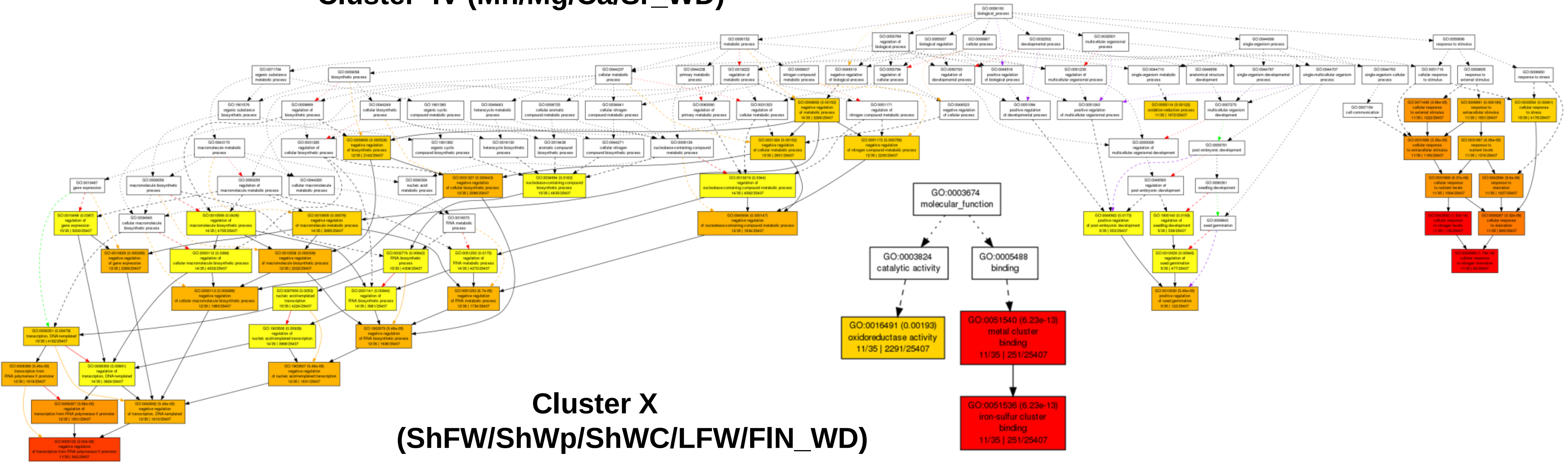

Cluster X (ShFW/ShWp/ShWC/LFW/FIN_WD)

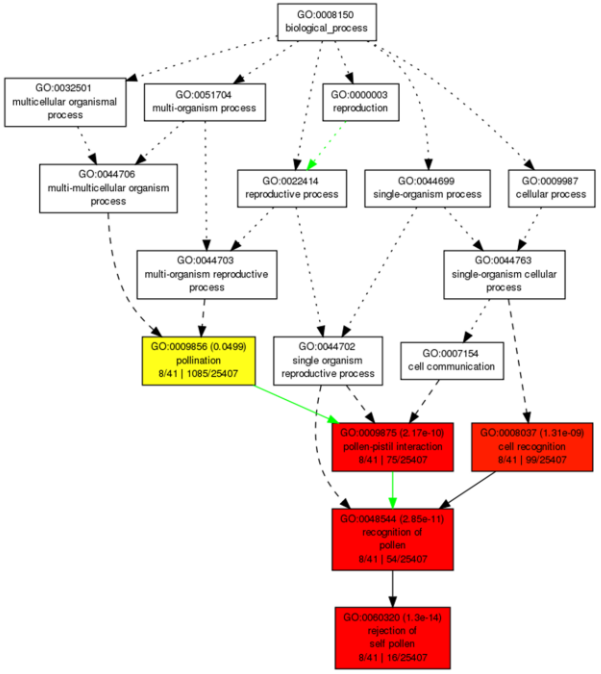

Cluster V (ZR/FlN_WD/ShFW/ShDW/ShWC/LFW/LDW_C)

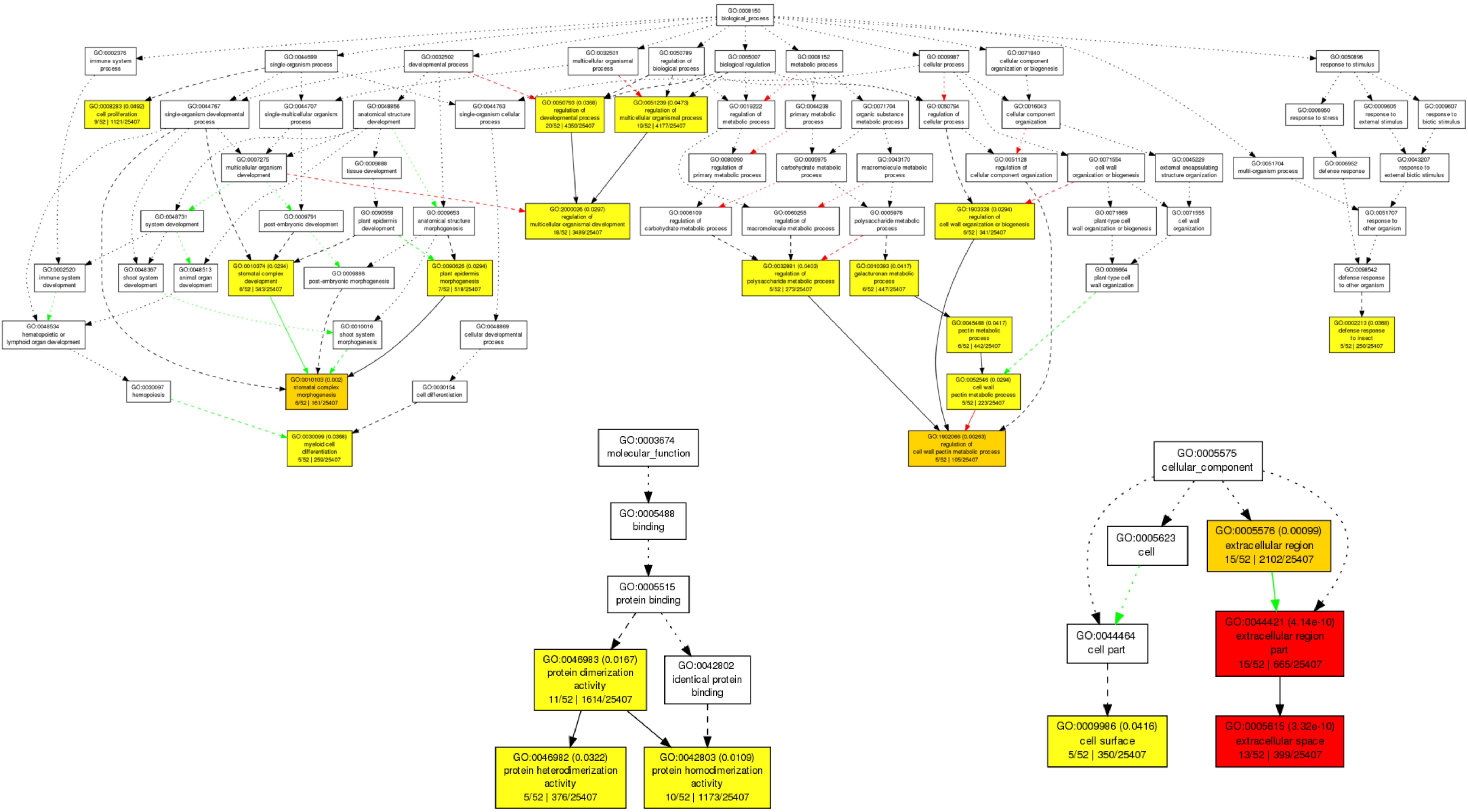

Cluster VII (Mg/Mn_WD)

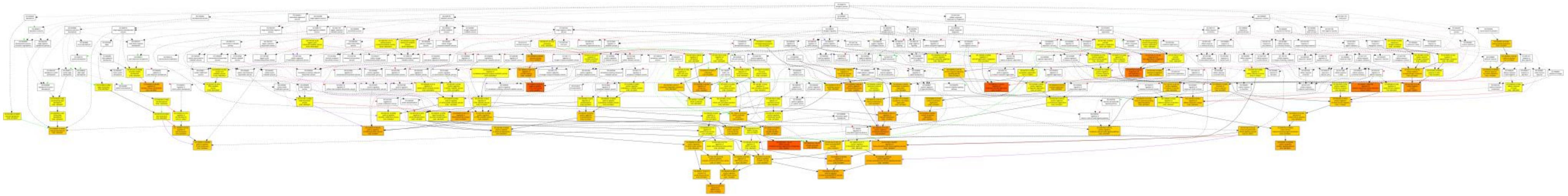

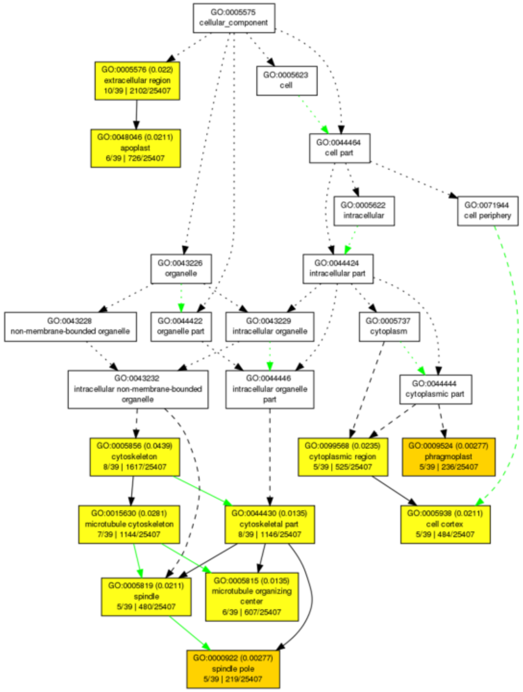

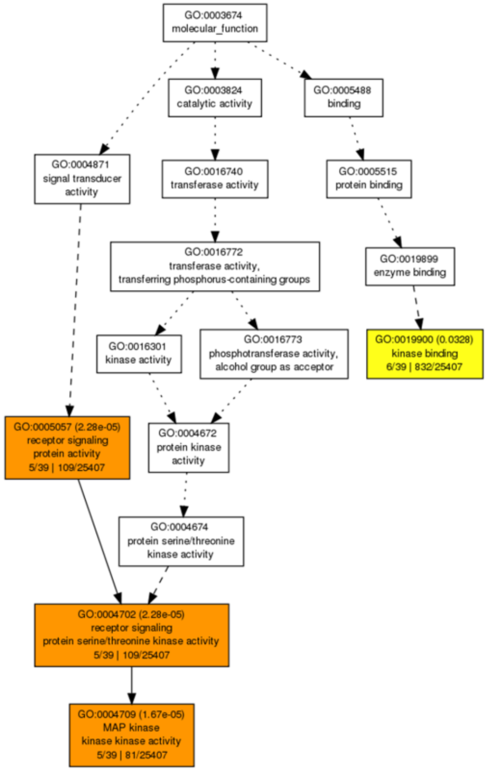

Cluster VIII (Ca/Sr/S_WD)

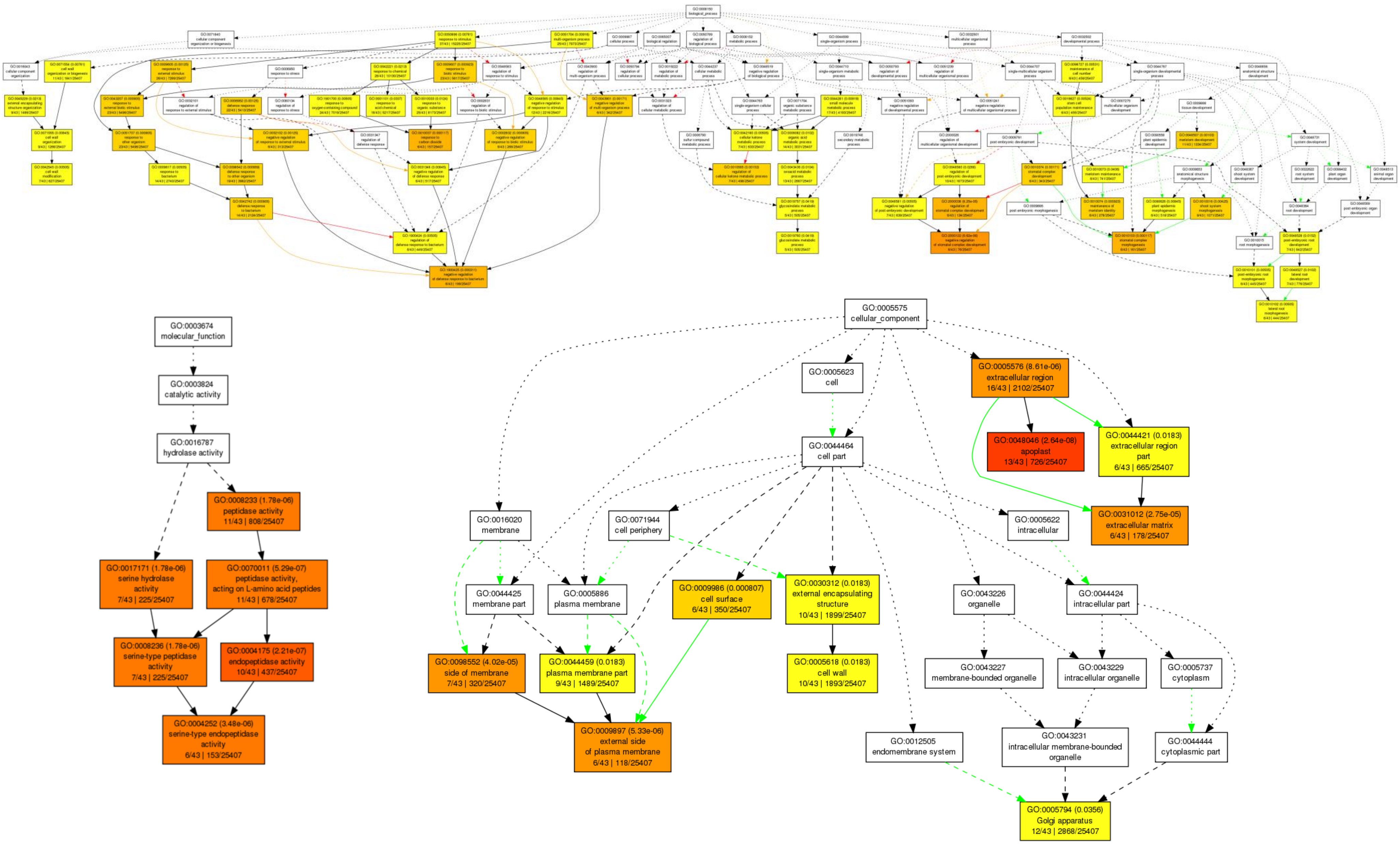

Table S1: *P*-values of significantly ($p<0.05$) different traits between controls Bol versus Bol/Bol under both watering regimes (C and WD). (+) means grafting has an increasing effect on the trait.

	***P*_C**		***P*_WD**	
**TotalShootFW**	0.0437	(+)		
**LeafDW**	0.0123	(+)		
**TotalShootDW**	0.0035	(+)		
**SPAD**	0.0189	(+)		
**ABA**			0.0007	(+)
**ZR**			0.0337	(+)
**JA**	0.0223	(+)		

Table S2: Pearson coefficients between significantly correlated traits ($p \leq 0.05$) for plants under control (_C) and water deficit (_WD). In bold, for each trait between irrigation regimes.

**Trait 1**	**Trait 2**	**r**	***p***
ZR_C	tZ_C	0.45	0.0000
ABA_C	tZ_C	0.42	0.0000
ABA_C	ZR_C	0.31	0.0005
JA_C	tZ_C	0.44	0.0000
JA_C	ZR_C	0.29	0.0012
JA_C	ABA_C	0.61	0.0000
B_C	ABA_C	0.28	0.0017
Ca_C	tZ_C	-0.25	0.0048
Ca_C	JA_C	-0.28	0.0018
Ca_C	B_C	0.27	0.0028
Cu_C	B_C	0.25	0.0053
Cu_C	Ca_C	0.78	0.0000
Fe_C	ABA_C	0.48	0.0000
Fe_C	B_C	0.51	0.0000
Fe_C	Ca_C	0.59	0.0000
Fe_C	Cu_C	0.71	0.0000
Mg_C	JA_C	-0.19	0.0341
Mg_C	B_C	0.46	0.0000
Mg_C	Ca_C	0.87	0.0000
Mg_C	Cu_C	0.67	0.0000
Mg_C	Fe_C	0.57	0.0000
Mn_C	tZ_C	-0.2	0.0302
Mn_C	JA_C	-0.21	0.0196
Mn_C	B_C	0.46	0.0000
Mn_C	Ca_C	0.85	0.0000
Mn_C	Cu_C	0.65	0.0000
Mn_C	Fe_C	0.63	0.0000
Mn_C	Mg_C	0.87	0.0000
Na_C	tZ_C	0.2	0.0299
Na_C	ZR_C	0.18	0.0411
Na_C	ABA_C	0.24	0.0078
Na_C	Ca_C	0.26	0.0036
Na_C	Cu_C	0.37	0.0000
Na_C	Fe_C	0.24	0.0087
Na_C	Mg_C	0.34	0.0001
Na_C	Mn_C	0.28	0.0021
P_C	tZ_C	-0.2	0.0301
P_C	ABA_C	0.21	0.0188
P_C	JA_C	-0.19	0.0369
P_C	B_C	0.53	0.0000
P_C	Ca_C	0.83	0.0000
P_C	Cu_C	0.67	0.0000

P_C	Fe_C	0.63	0.0000
P_C	Mg_C	0.86	0.0000
P_C	Mn_C	0.83	0.0000
P_C	Na_C	0.26	0.0043
Se_C	Cu_C	0.42	0.0000
S_C	tZ_C	-0.24	0.0078
S_C	ABA_C	0.2	0.0264
S_C	JA_C	-0.19	0.0360
S_C	B_C	0.51	0.0000
S_C	Ca_C	0.88	0.0000
S_C	Cu_C	0.69	0.0000
S_C	Fe_C	0.66	0.0000
S_C	Mg_C	0.9	0.0000
S_C	Mn_C	0.92	0.0000
S_C	Na_C	0.29	0.0010
S_C	P_C	0.91	0.0000
Sr_C	tZ_C	-0.27	0.0029
Sr_C	JA_C	-0.24	0.0080
Sr_C	B_C	0.38	0.0000
Sr_C	Ca_C	0.97	0.0000
Sr_C	Cu_C	0.73	0.0000
Sr_C	Fe_C	0.62	0.0000
Sr_C	Mg_C	0.92	0.0000
Sr_C	Mn_C	0.89	0.0000
Sr_C	Na_C	0.28	0.0020
Sr_C	P_C	0.88	0.0000
Sr_C	S_C	0.93	0.0000
Zn_C	ABA_C	0.37	0.0000
Zn_C	B_C	0.35	0.0001
Zn_C	Ca_C	0.81	0.0000
Zn_C	Cu_C	0.81	0.0000
Zn_C	Fe_C	0.73	0.0000
Zn_C	Mg_C	0.79	0.0000
Zn_C	Mn_C	0.75	0.0000
Zn_C	Na_C	0.33	0.0002
Zn_C	P_C	0.78	0.0000
Zn_C	S_C	0.8	0.0000
Zn_C	Sr_C	0.81	0.0000
LA_C	Fe_C	0.2	0.0279
LA_C	Zn_C	0.19	0.0401
FlN_C	B_C	-0.25	0.0045
FlN_C	P_C	-0.19	0.0319
FlN_C	Se_C	0.43	0.0000
FlN_C	LA_C	0.2	0.0261
LFW_C	ABA_C	-0.37	0.0000
LFW_C	JA_C	-0.31	0.0004
LFW_C	B_C	-0.27	0.0030
LFW_C	Se_C	0.34	0.0001

LFW_C	LA_C	0.35	0.0001
LFW_C	FlN_C	0.7	0.0000
ShFW_C	ABA_C	-0.35	0.0001
ShFW_C	JA_C	-0.31	0.0005
ShFW_C	B_C	-0.26	0.0043
ShFW_C	Se_C	0.33	0.0002
ShFW_C	LA_C	0.35	0.0001
ShFW_C	FlN_C	0.7	0.0000
ShFW_C	LFW_C	0.99	0.0000
LDW_C	ABA_C	-0.33	0.0002
LDW_C	JA_C	-0.24	0.0064
LDW_C	B_C	-0.23	0.0109
LDW_C	LA_C	0.24	0.0080
LDW_C	FlN_C	0.61	0.0000
LDW_C	LFW_C	0.88	0.0000
LDW_C	ShFW_C	0.86	0.0000
ShDW_C	ABA_C	-0.34	0.0001
ShDW_C	JA_C	-0.21	0.0172
ShDW_C	B_C	-0.3	0.0006
ShDW_C	Se_C	0.28	0.0016
ShDW_C	LA_C	0.28	0.0015
ShDW_C	FlN_C	0.71	0.0000
ShDW_C	LFW_C	0.91	0.0000
ShDW_C	ShFW_C	0.91	0.0000
ShDW_C	LDW_C	0.93	0.0000
SPAD_C	Se_C	-0.2	0.0298
ShWp_C	JA_C	-0.25	0.0056
ShWp_C	LA_C	0.26	0.0032
ShWp_C	LFW_C	0.38	0.0000
ShWp_C	ShFW_C	0.43	0.0000
ShWC_C	ABA_C	-0.35	0.0001
ShWC_C	JA_C	-0.32	0.0004
ShWC_C	B_C	-0.25	0.0053
ShWC_C	Se_C	0.33	0.0002
ShWC_C	LA_C	0.36	0.0001
ShWC_C	FlN_C	0.69	0.0000
ShWC_C	LFW_C	0.98	0.0000
ShWC_C	ShFW_C	1	0.0000
ShWC_C	LDW_C	0.85	0.0000
ShWC_C	ShDW_C	0.89	0.0000
ShWC_C	ShWp_C	0.45	0.0000
**ABA_WD**	**ABA_C**	0.31	0.0005
**Fe_WD**	**Fe_C**	0.36	0.0001
**FlN_WD**	**FlN_C**	0.37	0.0000
**LFW_WD**	**LFW_C**	0.46	0.0000
**ShFW_WD**	**ShFW_C**	0.44	0.0000
**LDW_WD**	**LDW_C**	0.41	0.0000
**ShDW_WD**	**ShDW_C**	0.44	0.0000

**SPAD_WD**	**SPAD_C**	0.26	0.0031
**ShWp_WD**	**ShWp_C**	0.29	0.0012
**ShWC_WD**	**ShWC_C**	0.44	0.0000
**B_WD**	**B_C**	0.27	0.0028
**Na_WD**	**Na_C**	0.38	0.0000
ABA_WD	ZR_WD	0.33	0.0002
JA_WD	ABA_WD	0.46	0.0000
B_WD	As_WD	0.36	0.0000
Ca_WD	ZR_WD	0.4	0.0000
Ca_WD	ABA_WD	0.19	0.0421
Ca_WD	B_WD	0.46	0.0000
Cr_WD	JA_WD	0.35	0.0001
Cr_WD	As_WD	-0.27	0.0031
Cr_WD	Ca_WD	-0.18	0.0440
Cu_WD	ZR_WD	0.37	0.0000
Cu_WD	ABA_WD	0.31	0.0005
Cu_WD	B_WD	0.5	0.0000
Cu_WD	Ca_WD	0.67	0.0000
Fe_WD	ZR_WD	0.41	0.0000
Fe_WD	ABA_WD	0.22	0.0147
Fe_WD	As_WD	0.22	0.0149
Fe_WD	B_WD	0.62	0.0000
Fe_WD	Ca_WD	0.7	0.0000
Fe_WD	Cu_WD	0.69	0.0000
K_WD	ZR_WD	0.36	0.0001
K_WD	ABA_WD	0.41	0.0000
K_WD	JA_WD	0.32	0.0004
K_WD	B_WD	0.3	0.0009
K_WD	Ca_WD	0.64	0.0000
K_WD	Cu_WD	0.61	0.0000
K_WD	Fe_WD	0.53	0.0000
Li_WD	As_WD	0.47	0.0000
Li_WD	B_WD	0.4	0.0000
Li_WD	Cr_WD	-0.34	0.0001
Mg_WD	ZR_WD	0.36	0.0000
Mg_WD	ABA_WD	0.23	0.0099
Mg_WD	B_WD	0.6	0.0000
Mg_WD	Ca_WD	0.87	0.0000
Mg_WD	Cu_WD	0.71	0.0000
Mg_WD	Fe_WD	0.67	0.0000
Mg_WD	K_WD	0.68	0.0000
Mg_WD	Li_WD	0.2	0.0315
Mn_WD	ZR_WD	0.37	0.0000
Mn_WD	B_WD	0.58	0.0000
Mn_WD	Ca_WD	0.94	0.0000
Mn_WD	Cu_WD	0.68	0.0000
Mn_WD	Fe_WD	0.72	0.0000
Mn_WD	K_WD	0.63	0.0000

Mn_WD	Mg_WD	0.93	0.0000
Mo_WD	B_WD	0.48	0.0000
Mo_WD	Cr_WD	0.47	0.0000
Mo_WD	Fe_WD	0.34	0.0002
Na_WD	ZR_WD	0.39	0.0000
Na_WD	ABA_WD	0.2	0.0254
Na_WD	B_WD	0.23	0.0112
Na_WD	Ca_WD	0.53	0.0000
Na_WD	Cu_WD	0.54	0.0000
Na_WD	Fe_WD	0.49	0.0000
Na_WD	K_WD	0.49	0.0000
Na_WD	Mg_WD	0.54	0.0000
Na_WD	Mn_WD	0.52	0.0000
P_WD	ZR_WD	0.29	0.0012
P_WD	ABA_WD	0.29	0.0015
P_WD	As_WD	0.18	0.0432
P_WD	B_WD	0.6	0.0000
P_WD	Ca_WD	0.82	0.0000
P_WD	Cu_WD	0.7	0.0000
P_WD	Fe_WD	0.63	0.0000
P_WD	K_WD	0.66	0.0000
P_WD	Li_WD	0.25	0.0065
P_WD	Mg_WD	0.87	0.0000
P_WD	Mn_WD	0.87	0.0000
P_WD	Na_WD	0.46	0.0000
Sb_WD	JA_WD	0.25	0.0057
Sb_WD	As_WD	-0.27	0.0030
Sb_WD	B_WD	0.23	0.0111
Sb_WD	Cr_WD	0.74	0.0000
Sb_WD	Fe_WD	0.22	0.0176
Sb_WD	Li_WD	-0.34	0.0002
Sb_WD	Mo_WD	0.61	0.0000
S_WD	ZR_WD	0.3	0.0008
S_WD	ABA_WD	0.23	0.0105
S_WD	B_WD	0.6	0.0000
S_WD	Ca_WD	0.88	0.0000
S_WD	Cu_WD	0.66	0.0000
S_WD	Fe_WD	0.63	0.0000
S_WD	K_WD	0.6	0.0000
S_WD	Li_WD	0.27	0.0028
S_WD	Mg_WD	0.9	0.0000
S_WD	Mn_WD	0.9	0.0000
S_WD	Na_WD	0.43	0.0000
S_WD	P_WD	0.91	0.0000
Sr_WD	ZR_WD	0.34	0.0001
Sr_WD	ABA_WD	0.23	0.0103
Sr_WD	B_WD	0.53	0.0000
Sr_WD	Ca_WD	0.97	0.0000

Sr_WD	Cu_WD	0.7	0.0000
Sr_WD	Fe_WD	0.7	0.0000
Sr_WD	K_WD	0.69	0.0000
Sr_WD	Mg_WD	0.92	0.0000
Sr_WD	Mn_WD	0.95	0.0000
Sr_WD	Na_WD	0.52	0.0000
Sr_WD	P_WD	0.87	0.0000
Sr_WD	S_WD	0.91	0.0000
Zn_WD	ZR_WD	0.37	0.0000
Zn_WD	ABA_WD	0.37	0.0000
Zn_WD	B_WD	0.51	0.0000
Zn_WD	Ca_WD	0.81	0.0000
Zn_WD	Cu_WD	0.72	0.0000
Zn_WD	Fe_WD	0.68	0.0000
Zn_WD	K_WD	0.68	0.0000
Zn_WD	Mg_WD	0.82	0.0000
Zn_WD	Mn_WD	0.82	0.0000
Zn_WD	Na_WD	0.43	0.0000
Zn_WD	P_WD	0.83	0.0000
Zn_WD	S_WD	0.83	0.0000
Zn_WD	Sr_WD	0.86	0.0000
LA_WD	ZR_WD	-0.22	0.0160
LA_WD	ABA_WD	-0.23	0.0124
LA_WD	Ca_WD	-0.2	0.0241
LA_WD	Fe_WD	-0.2	0.0267
LA_WD	Li_WD	-0.18	0.0471
LA_WD	Mn_WD	-0.21	0.0200
LA_WD	P_WD	-0.24	0.0092
LA_WD	S_WD	-0.19	0.0348
LA_WD	Sr_WD	-0.21	0.0201
FlN_WD	B_WD	-0.22	0.0170
FlN_WD	Cr_WD	-0.21	0.0235
FlN_WD	LA_WD	0.23	0.0091
LFW_WD	ABA_WD	-0.2	0.0264
LFW_WD	As_WD	0.18	0.0475
LFW_WD	LA_WD	0.41	0.0000
LFW_WD	FlN_WD	0.67	0.0000
ShFW_WD	ABA_WD	-0.2	0.0254
ShFW_WD	LA_WD	0.42	0.0000
ShFW_WD	FlN_WD	0.67	0.0000
ShFW_WD	LFW_WD	0.99	0.0000
LDW_WD	LA_WD	0.37	0.0000
LDW_WD	FlN_WD	0.67	0.0000
LDW_WD	LFW_WD	0.87	0.0000
LDW_WD	ShFW_WD	0.85	0.0000
ShDW_WD	B_WD	-0.22	0.0159
ShDW_WD	LA_WD	0.39	0.0000
ShDW_WD	FlN_WD	0.7	0.0000

ShDW_WD	LFW_WD	0.91	0.0000
ShDW_WD	ShFW_WD	0.9	0.0000
ShDW_WD	LDW_WD	0.96	0.0000
ShWp_WD	ABA_WD	-0.22	0.0157
ShWp_WD	Cu_WD	-0.2	0.0307
ShWp_WD	Fe_WD	-0.22	0.0144
ShWp_WD	Li_WD	0.32	0.0003
ShWp_WD	Sb_WD	-0.2	0.0291
ShWp_WD	LFW_WD	0.29	0.0012
ShWp_WD	ShFW_WD	0.34	0.0001
ShWp_WD	SPAD_WD	-0.18	0.0492
ShWC_WD	ABA_WD	-0.21	0.0216
ShWC_WD	LA_WD	0.41	0.0000
ShWC_WD	FlN_WD	0.66	0.0000
ShWC_WD	LFW_WD	0.98	0.0000
ShWC_WD	ShFW_WD	1	0.0000
ShWC_WD	LDW_WD	0.83	0.0000
ShWC_WD	ShDW_WD	0.89	0.0000
ShWC_WD	ShWp_WD	0.37	0.0000

Table S3: Correlations between principal components (1 and 2) and original traits.

Traits in C	CP 1	CP 2
ABA	0.28	-0.48
B	0.50	-0.32
Ca	0.92	0.17
Cu	0.82	0.12
Fe	0.76	-0.12
FlN	-0.06	0.69
iP	-0.11	-0.08
JA	-0.13	-0.45
LA	0.15	0.32
LDW	-0.03	0.89
LFW	-0.02	0.97
Mg	0.93	0.01
Mn	0.93	0.04
Na	0.32	-0.16
P	0.92	-0.03
S	0.96	0.01
Se	0.07	0.31
ShDW	-0.06	0.91
ShFW	-0.01	0.97
ShWC	-0.01	0.97
ShWp	0.12	0.32
SPAD	-0.01	-0.17
Sr	0.95	0.13
tZ	-0.16	-0.21
Zn	0.90	0.01
ZR	0.10	-0.26

Traits in WD	CP 1	CP 2
ABA	0.37	-0.12
As	0.14	0.26
B	0.65	-0.05
Ca	0.89	0.27
Cr	-0.03	-0.29
Cu	0.80	0.10
Fe	0.79	0.09
FlN	-0.17	0.73
JA	0.08	-0.17
K	0.73	0.10
LA	-0.31	0.34
LDW	-0.19	0.87
LFW	-0.26	0.93
Li	0.19	0.10
Mg	0.93	0.14
Mn	0.93	0.17
Mo	0.14	-0.23
Na	0.58	0.11
P	0.90	0.16
S	0.90	0.19
Sb	0.05	-0.27
ShDW	-0.23	0.90
ShFW	-0.28	0.93
ShWC	-0.29	0.92
ShWp	-0.18	0.20
SPAD	0.09	0.11
Sr	0.93	0.22
Zn	0.89	0.13
ZR	0.45	0.06

Table S4: QTLs included within each cluster or genomic region. In parenthesis, QTLs showing weaker linkage.

Cluster	QTLs							
**I**	FlN_WD_1	JA_C_1						
**II**	Mg_WD_2	Mn_WD_2	Zn_WD_2					
**III**	ShDW_DW_3	FlN_WD_3						
**IV**	Mn_WD_4	Mg_WD_4	Sr_WD_4	Ca_WD_4				
**V**	ZR_WD_4	FlN_WD_4.2	LFW_C_4	ShDW_C_4	ShFW_C_4	ShWC_C_4	LDW_C_4	ShWC_WD_4
**VI**	P_WD_6	B_WD_6	Ca_WD_6	Mg_WD_6	S_WD_6	Sr_WD_6	Zn_WD_6	
**VII**	Mg_WD_7	Mn_WD_7						
**VIII**	Ca_WD_8	S_WD_8	Sr_WD_8					
**IX**	(ZR_WD_9)	FlN_WD_9.1	Mn_WD_9	LDW_WD_9				
**X**	ShFW_WD_9	ShWp_WD_9	FlN_WD_9.2	LFW_WD_9	ShWC_WD_9			
**XI**	Mg_WD_10	Mn_WD_10	P_WD_10	(ZR_WD_10)				
**XII**	ABA_WD_5	ZR_WD_5						

Table S5: Summary list of candidate genes in drought tolerance QTL clusters, some segregating for frameshift Indels [48] in parental genomes, E9 or L5, (Mut.). The mRNA reference, its starting physical position in the chromosome (Start), its relative root expression (Exp.) in Heinz cultivar (Max: maximum, H: high, M: medium, VL: very low, L: low and N: no data), and the number of genes counted from the QTL peak (Ord.) are also shown.

Cluster	Mut	Exp	Start	Annotation	mRNA	Ord
IX		Max	990503	Galactosylgalactosylxylosylprotein 3-beta-glucuronosyltransferase 1	Solyc09g007420.2.1	95
IX		Max	1092950	Peroxidase 21	Solyc09g007520.2.1	85
IX	L5	N	1176696	MYB transcription factor (transcription factor SRM1-like)	Solyc09g007580.1.1	79
IX		Max	1212465	Fasciclin-like arabinogalactan protein 7	Solyc09g007650.1.1	72
IX		Max	1214595	Fasciclin-like arabinogalactan protein 7	Solyc09g007660.1.1	71
IX		Max	1295368	Aquaporin 2 (aquaporin PIP2-1)	Solyc09g007760.2.1	61
IX	E9/L5	Max	1300967	Aquaporin 2 (aquaporin PIP2-1)	Solyc09g007770.2.1	60
IX		Max	1332643	Auxin response factor 3	Solyc09g007810.2.1	56
IX		M	1401117	Manganese transporter mntH	Solyc09g007870.2.1	50
IX	E9, L5	Max	1413536	Phenylalanine ammonia-lyase	Solyc09g007890.1.1	48
IX		H	1607740	Membrane magnesium transporter 1	Solyc09g008140.2.1	23
IX		Max	1690129	ABC transporter ATP-binding protein/permease C9B6.09c	Solyc09g008240.2.1	13
IX		Max	1718596	MYB transcription factor 38 (protein blind-like1, bli1)	Solyc09g008250.2.1	12
IX		Max	1783996	Xyloglucan endotransglucosylase/hydrolase 12	Solyc09g008320.2.1	5
IX		Max	1921241	Phosphatidylinositol-4-phosphate 5-kinase 9	Solyc09g008480.2.1	0
IX	L5	Max	2036208	NCS1 family transporter (purine-uracil permease NCS1)	Solyc09g008550.2.1	7
IX		Max	2048866	1-aminocyclopropane-1-carboxylate oxidase	Solyc09g008560.2.1	8
IX		VL	2157113	Ethylene receptor	Solyc09g008720.1.1	22
IX		N	2449483	Gibberellin 20-oxidase	Solyc09g009110.2.1	57
IX		Max	2488251	D-mannose binding lectin family protein expressed	Solyc09g009150.1.1	61
IX		Max	2918891	Hydrolase alpha/beta fold family protein (6 copies in tandem)	Solyc09g009500.2.1	96
III		L	52192450	Membrane related protein (Lipid-binding START)	Solyc03g081320.2.1	41

III		M	52381445	G protein-coupled seven transmembrane receptor	Solyc03g082440.1.1	28
III	L5	Max	52393131	Leucine-rich repeat receptor-like protein kinase PEPR1	Solyc03g082470.2.1	25
III		Max	52438391	Auxin-responsive family protein	Solyc03g082510.1.1	21
III	L5,E9	M	52456478	Auxin responsive SAUR protein	Solyc03g082520.1.1	20
III	L5,E9	L	52461138	Auxin-responsive family protein	Solyc03g082530.1.1	19
III	L5	N	52636771	WRKY transcription factor 27	Solyc03g082750.1.1	0
III	L5	Max	52641646	Extensin-like protein Dif10 (Fragment)	Solyc03g082770.1.1	0
III		M	52861267	Protein ABIL1	Solyc03g083030.2.1	5
III		N	53164701	Response regulator 8	Solyc03g083340.1.1	36
III		M	53896143	Trehalose 6-phosphate phosphatase	Solyc03g083960.2.1	97
III		Max	53931600	Cortical cell-delineating protein (10 copies in tandem)	Solyc03g083990.1.1	100
X	L5	VL	68345675	EPIDERMAL PATTERNING FACTOR-like protein 4	Solyc09g082620.2.1	11
X	L5	VL	68348659	Acireductone dioxygenase	Solyc09g082630.2.1	10
X	L5	Max	68558897	Calcium-transporting ATPase 1	Solyc09g082880.1.1	0
X	L5	H	68630545	G protein gamma subunit 1	Solyc09g082940.2.1	0
X	L5	M	68643060	Pyruvate kinase	Solyc09g082970.2.1	0
X		VL	69089700	Proteinase inhibitor I (wound induced)	Solyc09g083430.1.1	0
X		VL	69123240	Chymotrypsin inhibitor-2 (wound induced)	Solyc09g084450.2.1	0
X	SNP	VL	69357833	Ethylene receptor ETR6	Solyc09g089610.2.1	0
X	L5	Max	69440018	1-aminocyclopropane-1-carboxylate oxidase-like prot.	Solyc09g089680.2.1	7
X	L5	VL	69445158	1-aminocyclopropane-1-carboxylate oxidase-like prot.	Solyc09g089690.2.1	8
X	L5	N	69448032	1-aminocyclopropane-1-carboxylate oxidase-like prot.	Solyc09g089700.2.1	9
X	L5	L	69448681	1-aminocyclopropane-1-carboxylate oxidase-like prot.	Solyc09g089710.2.1	10
X	L5	N	69450677	1-aminocyclopropane-1-carboxylate oxidase-like prot.	Solyc09g089720.1.1	11
X	L5	Max	69474948	1-aminocyclopropane-1-carboxylate oxidase-like prot.	Solyc09g089830.2.1	22
X	L5	N	69480316	1-aminocyclopropane-1-carboxylate oxidase-like prot.	Solyc09g089840.1.1	23
X		N	69519056	Ethylene responsive transcription factor 1a	Solyc09g089910.1.1	30
X		N	69522423	Ethylene-responsive transcription factor 5	Solyc09g089920.1.1	31
X		M	69543443	Ethylene responsive transcription factor 1a	Solyc09g089930.1.1	32

X		Max	69675472	R2R3MYB transcription factor 15 (SlMYB15)	Solyc09g090130.2.1	52
X		Max	69741894	Dehydration-responsive protein-like	Solyc09g090190.2.1	58
X	L5	Max	69863984	Phosphate transporter PHO1	Solyc09g090360.2.1	75
X		Max	69981424	Peptide transporter, NRT1/ PTR FAMILY 5.2-related	Solyc09g090470.2.1	86
X		VL	70104399	Zinc finger protein CONSTANS-LIKE 5	Solyc09g090650.2.1	104
X		Max	70204211	R2R3MYB transcription factor 79 (SlMYB79)	Solyc09g090790.2.1	118
X	L5	Max	70215131	Plasma membrane associated protein (PM19L)	Solyc09g090800.1.1	119
X		Max	70314836	Auxin responsive protein	Solyc09g090910.1.1	130
X		Max	70523221	Disease resistance response/ dirigent-like protein 16 like	Solyc09g091210.2.1	160
X		N	70787703	Salicylic acid carboxyl methyltransferase	Solyc09g091530.1.1	192
X		N	70794052	Salicylic acid carboxyl methyltransferase	Solyc09g091540.1.1	193
X		VL	70802564	Salicylic acid carboxyl methyltransferase	Solyc09g091550.2.1	194
X		Max	70907293	ABC transporter G family member 40 (ABA transporter)	Solyc09g091660.2.1	205
V		Max	63259221	WRKY transcription factor 7	Solyc04g078550.2.1	19
V		Max	63345069	WUSCHEL-related homeobox-containing protein 4	Solyc04g078650.2.1	9
V	L5	Max	63359490	Hydroxycinnamoyl transferase	Solyc04g078660.1.1	8
V	SNP	Max	63407131	Subtilisin-like protease (Phytaspase 2)	Solyc04g078740.2.1	0
V		Max	63744550	Gibberellin receptor GID1L2	Solyc04g079190.2.1	0
V	L5	M	63757430	Patatin-like protein 1	Solyc04g079240.2.1	0
V	L5	M	63888688	MYB transcription factor (MYB44-like)	Solyc04g079360.1.1	0
V	L5	VL	63894231	Serpin (Serine protease inhibitor)	Solyc04g079370.2.1	0
V	L5	Max	63945915	Proteinase inhibitor I4 serpin	Solyc04g079450.2.1	0
V	L5	Max	63947195	Serpin 3	Solyc04g079460.1.1	0
V	L5	Max	63948225	Serpin (Serine protease inhibitor)	Solyc04g079470.2.1	0
V	L5	Max	63950580	Serpin (Serine protease inhibitor)	Solyc04g079480.2.1	0
V	L5	Max	63952704	Serpin (Serine protease inhibitor)	Solyc04g079490.1.1	0
V	L5	Max	63953169	Serpin 4	Solyc04g079500.1.1	0
V		L	64129353	Inositol 5-phosphatase 4 (phytate synthesis)	Solyc04g079820.2.1	0
V		M	64566435	Dehydration-responsive family protein	Solyc04g080360.2.1	0

V		H	64780769	Inositol pentakisphosphate 2-kinase	Solyc04g080670.2.1	7
V		L	64891808	Cytokinin oxidase/dehydrogenase	Solyc04g080820.2.1	22
V		Max	65084026	Zinc finger protein CONSTANS-LIKE 3	Solyc04g081020.2.1	42
V		Max	65224973	Auxin response factor 5	Solyc04g081240.2.1	64
V		L	65260642	Auxin-induced SAUR-like protein	Solyc04g081250.1.1	65
V		Max	65494438	Thaumatin-like protein	Solyc04g081550.2.1	95
V	L5	Max	65663862	Exocyst complex component 6	Solyc04g081730.2.1	113
V	L5	M	65667993	Prostaglandin E synthase 2-like	Solyc04g081740.2.1	114
